# Modeling predator and prey hotspots: Management implications of baleen whale co-occurrence with krill in Central California

**DOI:** 10.1371/journal.pone.0235603

**Published:** 2020-07-07

**Authors:** R. Cotton Rockwood, Meredith L. Elliott, Benjamin Saenz, Nadav Nur, Jaime Jahncke

**Affiliations:** 1 Point Blue Conservation Science, Petaluma, CA, United States of America; 2 biota.earth, Berkeley, CA, United States of America; University of Fribourg, SWITZERLAND

## Abstract

As global ocean-bound commerce increases, managing human activities has become important in reducing conflict with threatened wildlife. This study investigates environmental factors determining abundance and distribution of blue whales (*Balaenoptera musculus*), humpback whales (*Megaptera novaeangliae*) and their prey (*Euphausia pacifica* and *Thysanoessa spinifera*) in central California. We provide insights into environmental drivers of the ecology and distribution of these species, model whale distributions and determine coincident hotspots of whales and their prey that will help decrease human threats to whales and protect critical feeding habitat. We developed separate predictive models of whale abundances (using negative binomial regression on count data) and krill abundance (using a two-part hurdlemodel combining logistic and negative binomial regressions) over a 14 year period (2004–2017). Variables included *in situ* surface and midwater oceanographic measures (temperature, salinity, and fluorescence), basin-scale climate indices, and bathymetric- and distance-related data. Predictions were applied to 1 km^2^ cells spanning the study area for May, June, July, and September during each of the 14 years of surveys to identify persistent distribution patterns. Both whales and krill were found to consistently use the northeast region of Cordell Bank, the Farallon Escarpment, and the shelf-break waters. The main identified blue whale hotspots were also krill hotspots, while co-occurrence was more limited and varied seasonally for humpback whales and krill. These results are valuable in identifying patterns in important areas of ecological interaction to assist management of whales. Areas north of Cordell Bank are of particular management concern since they overlap with the end of the San Francisco Bay northern shipping lane. Our findings can help decrease threats to whales, particularly in important foraging areas, by supporting implementation of vessel management and informing potential conflicts with other human uses.

## Introduction

Our oceans face increasing pressures from human activities, including commercial fishing and shipping [1–4]. Worldwide, marine vessel density has increased fourfold from the early 1990s through 2012 [5]. An increase in traffic in conjunction with other human activities (e.g. fisheries, energy production, climate change) places additional stress upon marine ecosystems, potentially resulting in loss of biodiversity and declines in endangered species [2,3]. Previous research has highlighted areas in central California within two National Marine Sanctuaries as important to foraging humpback (*Megaptera novaengliae*) and blue whales (*Balaenoptera musculus*) [6–8]. This high-use whale area near San Francisco Bay has seen numerous documented instances of ship strikes and 24 reported whale mortalities from 2008 to 2018. National Marine Sanctuaries protect ecological resources, including endangered whale populations. Modeling spatial patterns of habitat use by species within the Sanctuaries can help managers make more informed decisions to preserve a healthy, functioning ecosystem and recover depleted animal populations.

Coastal areas where eastern boundary currents occur, such as the California Current System, are characterized as nutrient-rich zones of high primary and secondary productivity [9]. These marine regions are made up of interlinking physical oceanographic processes and bathymetric features that host a diverse assemblage of marine birds and mammals. Productivity of California Current System waters varies temporally and spatially, predominantly driven by seasonal upwelling when coastal winds generate offshore Ekman transport which upwells nutrient rich deep water to the surface [9–11]. Some of the most biologically productive regions of the California Current System are near San Francisco Bay. Because of their biological significance, these areas have been designated as National Oceanographic and Atmospheric Administration sanctuaries, including the Greater Farallones (GFNMS), Cordell Bank (CBNMS) and Monterey Bay (MBNMS) National Marine Sanctuaries.

High levels of primary production in the California Current System support a marine food web that is dependent upon krill (order *Euphausiacea*). With a seasonally high biomass, krill are the main path of energetic transfer between primary producers and predators, including whales [12]. Two species dominate the euphausiid assemblage in the California Current System, *Euphausia pacifica* and *Thysanoessa spinifera*. *Euphausia pacifica* is the more abundant euphausiid [13] with densities an order of magnitude greater than *T*. *spinifera* [13,14]. Both species are found in high concentrations within the study region and associated with a variety of biological and physical factors. Diurnal movements of krill are primarily vertical [15]. Thus, factors affecting surface circulation (e.g. tidal influence, wind-driven upwelling, shear zones) can explain the majority of transport and accumulation of krill [e.g., 16].

Complex coastal and offshore bathymetry influences water dynamics which, in turn, aggregate non-motile and weak-swimming species forming prey hotspots [17–21]. For example, shelf-break and shelf-edge bathymetry create strong currents and advection that transport krill [22]. These oceanographic features are thought to create persistent krill aggregations or hotspots [19,22,23].

Over interannual to decadal timescales, basin-wide climate signals like El Niño Southern Oscillation (ENSO) and the Pacific Decadal Oscillation (PDO) affect winds, water temperature, salinity and their gradients which then affect the primary producers that krill depend on. The interplay of these cycles means seasonal to decadal variability in conditions can be significant in the California Current System. Increased sea surface temperature and decreased salinity in El Niño years lead to reductions in offshore zooplankton biomass [24–26], and the PDO has a documented strong effect of reducing California Current System zooplankton composition and biomass [27].

The distribution and abundance of krill help identify important foraging areas for marine predators. However, krill aggregations tend to be spatially (tens of meters to kilometers) and temporally (hours to days) ephemeral [28,29]. Krill predators continuously search for predictable dense pockets of their energy-rich prey, so only longer term analyses at finer temporal and spatial scales can reveal patterns of predator hotspots and how these may change across seasons and years.

The Federally-endangered blue whale migrates through the California Current System and exclusively consumes krill. Waters along the California coast are valuable to these migratory, highly specialized apex predators [30], and their presence is largely driven by the presence or absence of their prey [31–33]. Blue whales have great energetic demands, consuming up to two tons of krill daily [34–36]. The Eastern Pacific population of humpback whales was recently divided into two distinct population segments. The Central American distinct population segment is listed as Endangered, migrates between breeding grounds off Central America and feeding grounds off California and Oregon and was estimated to number 411 individuals based on 2004–2006 data [37]. The Mexican distinct population segment (~2,500 individuals) is listed as threatened and contributes the majority of the remaining whales migrating along the U.S. west coast and ranges from California to British Columbia during their migration [38].

In June 2013, the layout of San Francisco Bay shipping lanes was modified to primarily increase maritime safety and secondarily reduce the co-occurrence of whales and ships. While the overall footprint of shipping has decreased, the new design concentrates vessel traffic through areas important to foraging and migrating whales [6]. At the same time, in recent years, the number of entangled whales found off San Francisco Bay has increased drastically, likely due to heightened overlap with Dungeness Crab gear [39]. The combination of shipping and fishery impacts has made the region off San Francisco Bay one of significant concern for blue and humpback whales.

When predator and prey hotspots overlap with human use, as occurs off San Francisco Bay, there is potential for negative interactions. Information about the distribution of exposed species, the ecological drivers of those patterns and how they may differ between species can help formulate effective management plans. Here, we develop predictive models to better understand spatio-temporal patterns in blue whale, humpback whale and krill abundance in central California. These models provide a robust basis for recommendations to reduce the likelihood of whale strikes and entanglements. We constructed predictive spatio-temporal models using 14 years of acoustic prey data and visual whale observations in central California, combined with in situ oceanographic measures, bathymetric features, and large-scale climate indices. Using modeled predictions of abundance, we (1) identified environmental factors contributing to krill habitat use, (2) identified environmental drivers of blue and humpback whale occurrence and how they differ among months and years, (3) identify patterns in hotspot use and trophic overlap between blue and humpback whales and krill, and (4) discuss the implications that foraging hotspots have for management of vessel traffic and fishery bycatch.

## Materials and methods

### Study area and survey design

**Fig 1:**
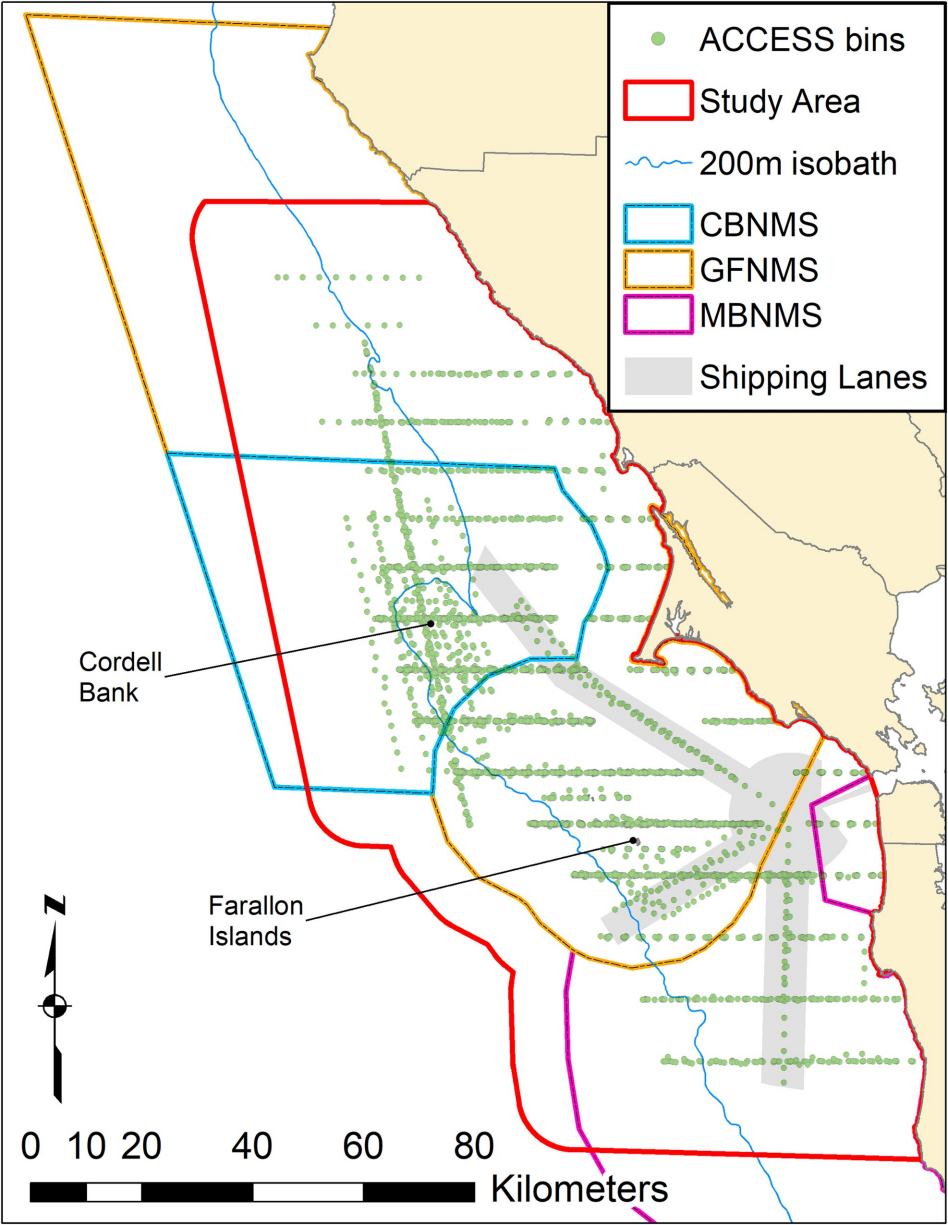
Study area. Study area within the Greater Farallones (GFNMS), Cordell Bank (CBNMS) and Monterey Bay National Marine Sanctuaries (MBNMS), California, USA, showing ACCESS program bin centers for all cruises as well as the San Francisco shipping lanes and the 200 m isobath.

We used data collected by the Applied California Current Ecosystem Studies (ACCESS) program (www.accessoceans.org), an ongoing collaboration between Point Blue Conservation Science, CBNMS, and GFNMS (Fig 1). Surveys were conducted 3–4 times each year from 2004–2017, during April to October, excluding August. These months were chosen to encompass the California Current System upwelling period; August was excluded due to resource limitations. ACCESS surveys consisted of transect lines that are oriented east-west through the Sanctuaries between Gualala, CA (38.3°N) and Pescadero, CA (37.2°N). The transects cross various bathymetric features including the continental shelf, shelf break (defined here as the 200 m isobath) and slope defined by the 50 to 1000 m isobaths. No permits were required to conduct this research because methods do not involve close interaction with marine mammals.

The ACCESS surveys simultaneously record counts of marine birds and mammals, surface and water-column oceanographic variables, and continuous hydroacoustics to estimate krill biomass. The observation effort (kilometers of trackline surveyed) varied by cruise and year, primarily as a function of time constraints and weather conditions. Temperature, salinity, and chlorophyll-*a* fluorescence were sampled vertically using a Conductivity-Temperature-Depth (CTD) profiler at permanent stations, and using a thermosalinograph while underway during transects. All data collected on transects (whale observations, krill acoustics and thermosalinograph measurements) were grouped in 3 km segments, or “bins”, a spatial scale previously documented as appropriate for modeling fine-scale patterns of marine megafauna habitat use [20,40]. Each bin’s centroid was subsequently assigned corresponding *in-situ* transect and remotely collected environmental data. End-of-line bins (<1 km in length) were discarded to avoid introducing errors [6,41,42].

### Oceanographic variables and generated predictive surfaces

Oceanographic variables of temperature, salinity, and chlorophyll-*a* fluorescence were recorded vertically *in situ* using a Sea-Bird Electronics SBE 19*Plus* SEACAT CTD profiler equipped with a WETStar fluorometer. Variables were sampled continuously from surface to maximum depth and the mean was taken for measurements from 1–9 m (surface variables) and for the 30–40 m depth range (midwater variables). The two chosen categories span the range of primary productivity in the photic zone and extend over the entire study area. The midwater depths (30–40 m) represent depths through which the thermocline ranges during different seasons and years [43]. Thermocline depth is related to krill abundance and distribution through effects on primary productivity and changes in shelf retention due to upwelling-driven cross-shore advection [16,44]. Because of these effects on krill, we expected that including a midwater category could improve predictions, as shown in previous modeling [6].

Underway measurements (used for surface variables in conjunction with the CTD cast data described above) were taken with a ship-mounted flow-through Turner Designs SCUFA fluorometer and a Sea-Bird Electronics SBE 38 CTD. While measured fluorescence is only a relative indicator of chlorophyll-*a* concentration, several sampling features help mitigate the effects of light exposure and temperature on the relationship between our fluorescence measures and chlorophyll-*a*. First, fluorescence measurements were corrected for temperature using established correction factors developed by Turner Designs. Second, the flow-through nature of the SCUFA allows light acclimation during the passage of phytoplankton from the ship’s hull through the water collection system to the SCUFA.

Since the environmental predictors were recorded along the vessel track (surface variables) and at oceanographic stations (midwater variables), we needed to interpolate them across the study area in order to create a prediction surface. To accomplish this, we created a prediction matrix of 1 km^2^ cells covering the study area.

Within surface and midwater categories, we created raster surfaces of temperature, salinity and fluorescence for each cruise (*n* = 246 surfaces; three variables at two depths for 41 cruises) on the 1 km^2^ prediction matrix using ordinary kriging interpolation with optimization. Kriging was chosen because it is based on statistical models that include autocorrelation assessments, thereby avoiding overemphasizing data along transect lines [45,46]. Parameter optimization was employed to standardize the interpolation process to minimize the mean square error for predictions, with the assumption that data were isotropic (not directionally dependent); measured locations were weighted by applying a default search radius. To ensure our use of isotropic kriging was appropriate, we used ArcGIS’s kriging tools to determine if an anisotropic search radius improved kriging metrics. We found no strong evidence of anisotropy, however, likely due to the relatively small study area and very dynamic oceanographic processes.

Common measures of the accuracy of kriging outputs include root mean squared error (RMSE), mean error (ME), and comparisons to the range of the measured data using leave-one-out cross validation [47,48]. To ensure the quality of the kriging output, we checked that 1) more than 90 percent of predictions were within the 95% CI of the measured data, 2) more than 90 percent of predictions were within 2 standard deviations of the mean of measured values, 3) percent RMSE was less than 30, and 4) the percent ME was less than 2. Kriging models that did not meet these metrics (*n* = 43) were de-trended in ArcGIS’s Trend Analysis program using second order trend removal and re-kriged until all quality metrics were met. We then predicted each oceanographic variable to the transect bins and model grid for each cruise.

### Climate indices

We included 4 oceanographic climate variables as potential model predictors: 1) PDO which is the first dominant mode of variability in sea surface temperature north of 20°N [49]; 2) North Pacific Gyre Oscillation (NPGO) which is the second dominant mode of variability in sea surface height in the northeast Pacific [50]; 3) Southern Oscillation Index (SOI) which is a standardized index based on the observed sea level pressure differences between Tahiti and Darwin, Australia [51]; and 4) Upwelling Index (UI) which is the amount of water upwelled from the base of the Ekman layer per 100 m of coastline (Table 1). We used an average of two UI values along the California coastline to best match the study region (36°N 122°W and 39°N 125°W). The UI is a regional index, while the other three indices represent ocean-basin scale patterns. Monthly mean values of all variables were matched with cruise timing. We included climate lags in modeling efforts to examine the influence of oceanic conditions up to 3 months prior to each cruise month.

**Table 1:** Description and ranges of variables used to model krill distribution and blue and humpback whale abundance. All variables used in both modeling exercises with exception of effort (* used only in krill analysis) or detection (** used only in blue whale analysis) variables.

Variable	Description	Mean ± SD	Min–Max values	CV
***Oceanographic Variables***				
Temperature, surface	Average surface (1–9 m) temperature (°C)	12.57 ± 2.06	8.2–18.55	0.16
Temperature, midwater	Average midwater (30–40 m) temperature (°C)	10.24 ± 1.11	7.99–14.95	0.11
Salinity, surface	Average surface (1–9 m) salinity (psu)	33.43 ± 0.33	29.88–34.04	0.01
Salinity, midwater	Average midwater (30–40 m) salinity (psu)	33.62 ± 0.26	32.63–34.08	0.01
Fluorescence, surface	Average surface (1–9 m) fluorescence (mg / m^3^)	9.1 ± 10.2	0.03–58.87	1.12
Fluorescence, midwater	Average midwater (30–40 m) fluorescence (mg / m^3^)	3.19 ± 3.68	0.14–26.1	1.15
***Bathymetric Variables***				
Distance to mainland	Distance from bin midpoint to nearest mainland feature (km)	25.15 ± 11.09	1.21–50.46	0.44
Distance to island	Distance from bin midpoint to nearest island feature (km)	27.25 ± 17.49	0.06–108	0.64
Distance to Cordell Bank	Distance from bin midpoint to nearest edge of Cordell Bank (km)	38.54 ± 24.35	0.81–115.47	0.63
Distance to 200 m isobath	Distance from bin midpoint to 200 m isobath (km)	12.74 ± 11.21	0.02–47.3	0.88
Average depth	Average depth at bin midpoint (m) (California Department of Fish and Wildlife)	168.73 ± 224.51	7.18–2075.63	1.33
Contour index	Contour index: (max depth—min depth)/max depth (unitless) (California Department of Fish and Wildlife)	22.87 ± 20.47	0–100	0.90
***Climate Indices (present month up to 3-month lag)***				
SOI	Monthly Southern Oscillation Index value (http://www.cgd.ucar.edu/cas/catalog/climind/soi.html)	0.11 ± 1.77	-6.7–4.3	
PDO	Monthly Pacific Decadal Oscillation value (http://jisao.washington.edu/pdo/PDO.latest)	0.04 ± 1.16	-2.21–2.35	
NPGO	Monthly North Pacific Gyre Oscillation value (http://eros.eas.gatech.edu/npgo/)	0.21 ± 1.08	-2.1–2.23	
UI	Averaged Upwelling Index value (36°N 122°W and 39°N 125°W) (http://www.pfeg.noaa.gov/products/PFEL/modeled/indices/upwelling/NA/data_download.html)	190.99 ± 81.1	2–389.5	0.42
***Effort- or detection bias-related variables***				
Cell Count *	Vertical cells, for which krill were summed (unitless)	266 ± 158	1–741	0.60
Bin Size **	Area of bin surveyed (ln(km^2^))	2.75 ± 0.66	0–3	0.24

### Bathymetric and distance variables

For each observation point along cruise transects and each 1 km^2^ prediction cell midpoint, we calculated water depth and distances to important land and ocean features. Key features included mainland, Cordell Bank, nearby islands in the Farallon islands archipelago, and to the 200 m isobath (or shelf-break). The shortest distance to each of these features was calculated in ArcGIS (10.2, ESRI Redlands, CA) from the midpoint of each 3 km bin or 1 km^2^ prediction cell. For each bin and cell, depths were calculated in ArcGIS using a bathymetric surface (California Department of Fish and Wildlife) and we generated a contour index–a metric of ocean floor roughness–using methods outlined in Yen *et al*. [20].

### Acoustic krill biomass

Zooplankton were sampled acoustically every 2 s along transects using a Simrad EK60 echosounder sampling at 38, 120 and 200 kHz. Raw volume backscatter data were post-processed and integrated into 200 m width by 5 m depth bins using Echoview software (Sonardata, Pty. Ltd.). To avoid surface and bottom interference, backscatter from 0–5 m and from depths <2 m from the bottom were excluded. The level of noise reduction was adjusted by visually comparing echograms until noise at depth appeared equal [52] and time-varied noise was subtracted from each echogram before integration into bins [53].

We estimated target strengths (the ratio of reflected to transmitted sound wave intensity) for krill using the Stochastic Distorted-Wave Bourne Approximation [54–57]. We used the Stochastic Distorted-Wave Bourne Approximation for Antarctic krill by Demer & Conti [56] to estimate target strengths for 8–30 mm length krill, roughly spanning the size of juvenile, immature, and adult stages for *E*. *pacifica* and *T*. *spinifera*.

Decibel differencing and echogram classification was conducted according to methods detailed in Manugian *et al*. [53]. Krill biomass (g/m^2^) was calculated by first apportioning the acoustic backscatter to a length-frequency distribution of individual krill determined from trawl surveys concurrent to acoustic sampling. The backscatter was divided by the corresponding individual backscattering cross sections and then multiplied by a length-weight relationship to obtain the mass of krill [52]. The length-weight relationship was derived from sampled individual krill *M*_*L*_ = 0.0002*L*^2^–0.0017*L + 0*.*005*, where *L* is an individual krill length (mm). Acoustic data were integrated throughout the water column to 200 m depth and binned to 3 km bins to match other datasets (*n* = 5,801; maximum biomass: 34,699 g).

### Blue and humpback whales

Marine mammal at-sea surveys were conducted during each ACCESS cruise from the vessel’s flying bridge. Standardized ship-board line-transect methods [58] were used to count whales while “on effort”, which was defined as: daylight hours while the vessel was underway at 10 knots [11]. During all cruises, one experienced observer was stationed on each side of the vessel and searched for animals using a combination of the unaided eye and 7 x 50 handheld binoculars while a third observer and data recorder searched from a central position. The total number of whales counted along each transect line was summed for each 3 km bin and assigned to each bin’s midpoint. Of 5,801 total bin counts, there were 179 blue whale presences (3.1%) and 930 humpback whale presences (16.0%).

### Krill modeling

All modeling was conducted in R version 3.6.1 [59]. We used Generalized Linear Modeling (GLM) in package *pscl* (*v*. 1.5.2) to predict distribution and abundance of krill using a two-part (hurdle) model [60]. The first part was a logistic regression model estimated for probability of observing a positive value vs. zero (3,722 positives; 996 zeros). The second part was a negative binomial model that was conditional on a positive value in the first part of the model. A hurdle model was chosen over an equivalent zero-inflated model because we believe there are no structural zeros (zero counts resulting from an underlying process separate from the sampling). We used log-transformed total volume of acoustically-sampled cells within each bin as an offset term to account for variable survey effort.

We conducted univariate testing of variables of interest (Table 1) for both parts of the two-part model; a full model was generated for krill including the most significant explanatory relationship of each variable for both parts of the model. Depending on the variable, we employed three univariate testing strategies to select most significant relationships for inclusion in multi-variable models. For oceanographic and bathymetric variables, we chose either a linear, quadratic, cubic or quartic relationship. Variables were included in the starting model at the highest order for which they were significant (*p*<0.05) in univariate fits. For distance variables, we tested 4 potential relationships (linear, quadratic, logarithmic, or inverse logarithmic) and chose the most significant relationship to krill. For climate indices, we selected either a no-, 1-, 2- or 3-month lag index. We included all selected variables in a full model and used manual backwards stepwise removal, ensuring that AIC values decreased at each step until all variables included in both parts of the final two-part model were significant (*p*<0.05). We then calculated AIC and BIC for all candidate models from each step. If the lowest AIC value was more than 2 units lower than the next model, we selected that model for the final model. When multiple models were identified using AIC (were within 2 ΔAIC units), the lowest BIC was used to select a single model.

Once we generated the final model, we tested potential interactions between year and specific variables that were in in both the logistic and the negative binomial parts of the two-part model based on *a priori* knowledge. Interactions with year were tested for static features (distance to the 200 m isobath, distance to land, distance to island, distance to Cordell Bank, contour index and average depth) and we built models with significant interactions using the likelihood ratio to test for improvement over models without an interaction. We validated model prediction capability using k-fold cross-validation (k = 10, 20 runs each) [53]. Each of the subsets was used as a “test” dataset and the other nine were combined and used as a “training” dataset; the procedure was repeated 20 times and we determined the root mean squared error (RMSE), mean absolute error (MAE) and significance of the cross-validated models. Variance inflation factor testing for multicollinearity was conducted among the linear portion of final model variables in each part of the two-part model. To examine spatial autocorrelation of the model, we calculated the Moran’s I statistic on the model residuals as well as creating maps of residuals to visually check for spatial patterns.

### Whale modeling

We used a GLM with a negative binomial distribution and log link (package *MASS v*. 7.3–51.4) to predict blue and humpback whale encounter rate (animals encountered per track segment). The modeling selection process followed the guidelines outlined in the krill section with a final model threshold of *p*<0.05 (for variable retention) and using an offset of log-transformed bin survey area to account for effort. We also compared the selected model to a zero-inflated GLM to determine if a zero-inflated model improved fit by addressing potential zeros resulting from whales being below the surface and unavailable for detection. In addition to the predictors in the negative binomial portion of the model, we tested variables affecting detection probability (swell, visibility, sea state) for inclusion in the zero-inflation portion of the models. We used the Vuong test to compare the negative binomial models with their zero-inflated counterparts. Based on the Vuong test results at p < 0.05, none of the zero-inflated models showed statistical improvement, hence we did not use zero-inflated negative binomial.

We computed the track segment-specific density as:

$$D_{i}=\frac{n_{i}}{2\times L_{i}\times ESW_{i}\times \text{g}{\left(0\right)}_{i}}$$

where *n* is the number of sightings for transect segment *i*, *L*_*i*_ is the segment length (km), ESW is the effective strip half-width (km), and g(0) is the probability of detection on the transect. ESW is also 1/f(0), where f(0) is the detection probability density function evaluated at zero perpendicular distance. To adjust for variability in detection of whales with different survey conditions, we used the same approaches as previous density estimation modeling [46,61,62]. Following Becker *et al*. [62], we used track segment-specific ESW and g(0) values (see S1 File). However, since our study area is much more restricted in size and has unique observation conditions (e.g., dense fog associated with calm waters) compared to the broader eastern Pacific, we estimated new g(0) and ESW values from ACCESS data rather than using values from Barlow *et al*. [63] and Barlow [64] (See S1 File).

### Blue whale, humpback whale and krill, hotspots and overlap

We predicted blue and humpback whale abundance and krill biomass to the 1 km^2^ prediction matrix using the final models. Cruises for which there was only 1 or two lines of oceanographic CTD data were excluded (*n* = 4). We created average monthly and yearly map sets for each separate species. Monthly calculations included 2004–17 while annual values included May through September, excluding August. We did not predict for April or October because these months were not consistently sampled. It is important to note that months and years were not sampled equally due to logistics. This has the potential to bias the monthly and yearly averages. However, where fewer months were sampled in a year, those months were balanced between higher- (July and September) and lower- (May and June) abundance months, which partially mitigates the effect of uneven sampling. In addition, monthly averages followed closely with seasonal patterns found in previous research [34,65]. We also created an all-inclusive (all years, all months) map for each species’ predicted hotspots using all predictions. For this map, we first averaged all available monthly predictions within each year, then averaged across years to equally weight all years despite the inconsistent number of months sampled within years. All maps were created in ArcMap 10.5 and map sets were scaled as percentiles of predicted values from the full predicted monthly or annual dataset for the particular species to allow for comparison between time periods.

For each month and grid cell we calculated the Getis-Ord G_i_* statistic, which identifies spatial clustering [66]. Significant positive G_i_* z-scores indicate spatial clustering of high values, in our case indicating species hotspots. Following Caldas de Castro and Singer [66], we used false discovery rate to correct the problem of multiple comparisons influencing the determination of significance for G_i_* values. False discovery rate is an alternative to overly conservative multiple-comparison corrections such as the Bonferroni method, which control the family-wise error rate. Instead, false discovery rate estimates and minimizes the proportion of false declarations of significance which ensures identification of as many significant clusters as possible [66]. Using the p-values produced using false discovery rate correction [67], we masked each raster according to significance and z-score sign, leaving only cells with positive z-scores and p-values less than 0.05. This created a set of monthly z-score rasters showing density hotspots of each species. Because we were interested in the relative use of space across time, rather than identifying hotspots due to differences in monthly abundance, we rescaled the z-scores for each raster to vary from zero to one. We then summed all rasters to produce a map for each species that represents the intensity and persistence of hotspot use across our study period. The values or the resulting summed surface can range from 0 (never classified as a hotspot) to 39 (classified as the top hotspot for all 39 modeled months).

To visualize overlap between each whale species and krill, we calculated the AB ratio for each month. In the AB ratio, the numerator represents the average product of the differences between the concentrations of predators and prey and their respective mean concentration, and the denominator is the product of the average concentrations of predators and prey [68]. AB ratio measures the increase in predator productivity that can be attributed to spatial overlap with prey, making it a good way to examine the trophic implications of fine-scale overlap [69]. We calculated the global AB ratio across the entire study region for each year to determine how trophic overlap varies through time. For each modeled month, we also calculated a local AB ratio for each raster cell and then took the mean and standard deviation of all AB ratio values across time. Maps of the mean indicate average trophic importance across the study period and maps of the standard deviation show the degree of AB ratio variability between months.

## Results

### Krill habitat association and predicted distribution

Significant variables in the logistic portion of the krill model (which predicted presence/absence) included all midwater oceanographic variables, distance to bathymetric features (mainland, island and 200 m isobath), contour index, and 4 climate indices (SOI, PDO, NPGO, UI); all the above were significant at *p*<0.05 (S2 and S3 Tables). No problematic multicollinearity was found between covariates of the final model. Variance inflation factor was less than 10 for all variables and below 3 for all non-interaction variables. Significant variables in the model which predicted krill abundance (part 2 of the model) included fluorescence at both surface and midwater depths but only midwater salinity, distance to bathymetric features (mainland, island, Cordell Bank), depth, contour index and four climate indices (SOI, PDO, NPGO, UI) (S2 and S3 Tables). The final krill model included an interaction in the count portion of the model between year and contour index; none of the other 4 variables examined demonstrated significant interaction with year.

**Fig 2:**
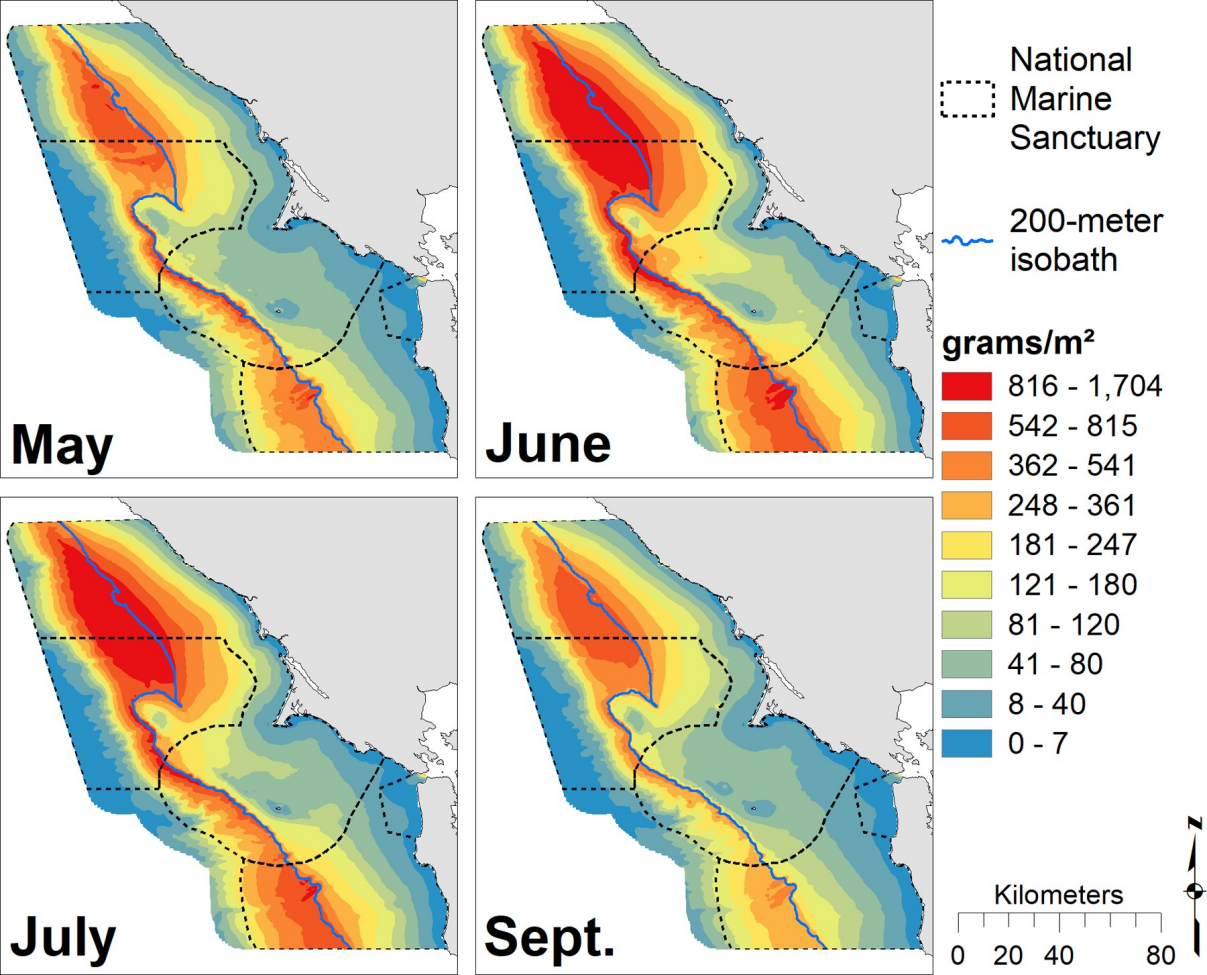
Monthly krill biomass. Predicted krill biomass for May (predictions averaged over n = 8 years), June (n = 7), July (n = 7), and September (n = 8); each gradation represents a decile. The 200 m isobath and boundaries of National Marine Sanctuaries included in blue and dashed black, respectively.

**Fig 3:**
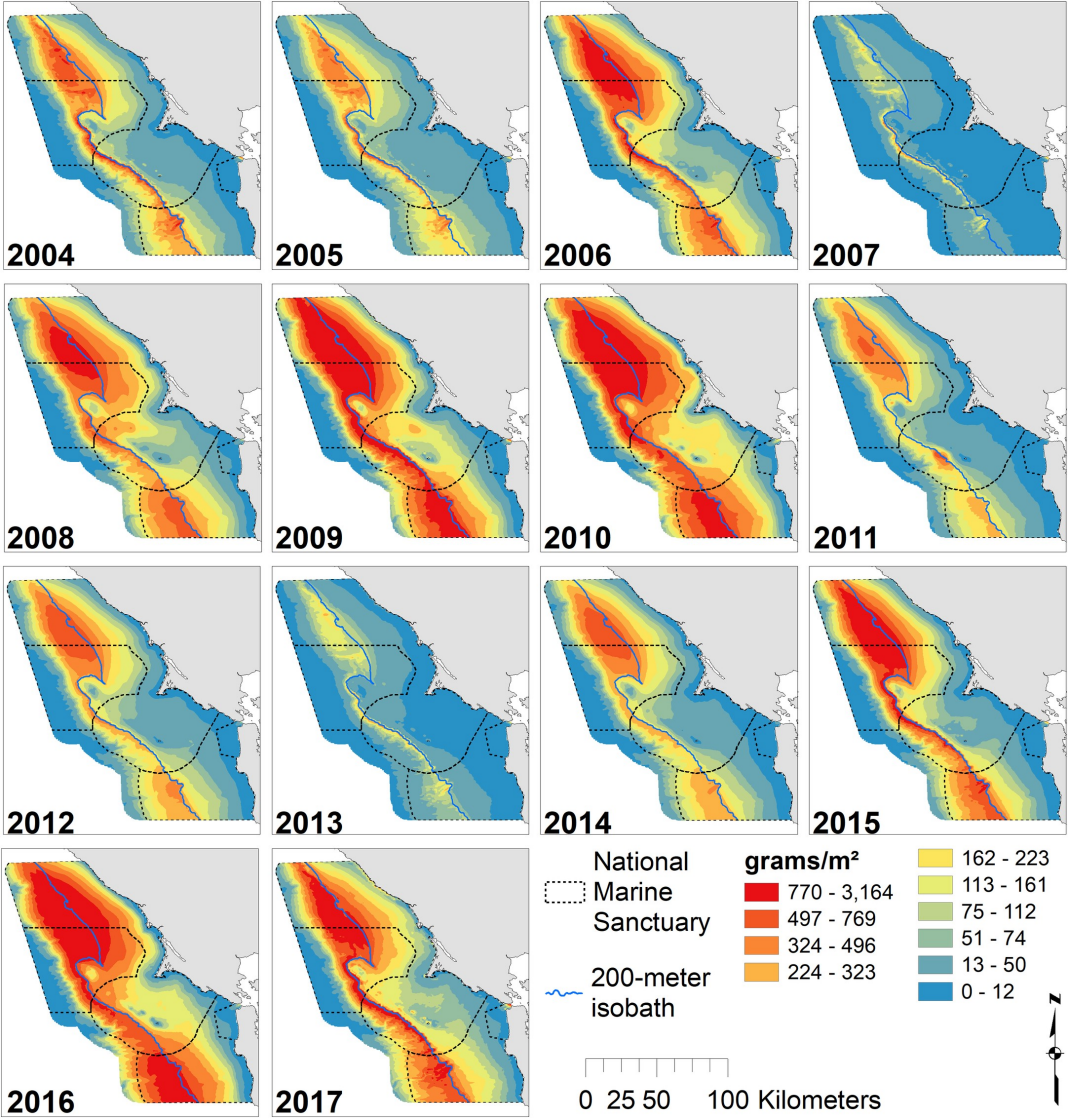
Annual krill biomass. Predicted krill biomass, 2004–2017: variations in annual modeled habitat use (predictions averaged over n = 3 months except 2010, n = 4; 2013–14 and 2016–17, n = 2); Legend as in Fig 2.

**Fig 4:**
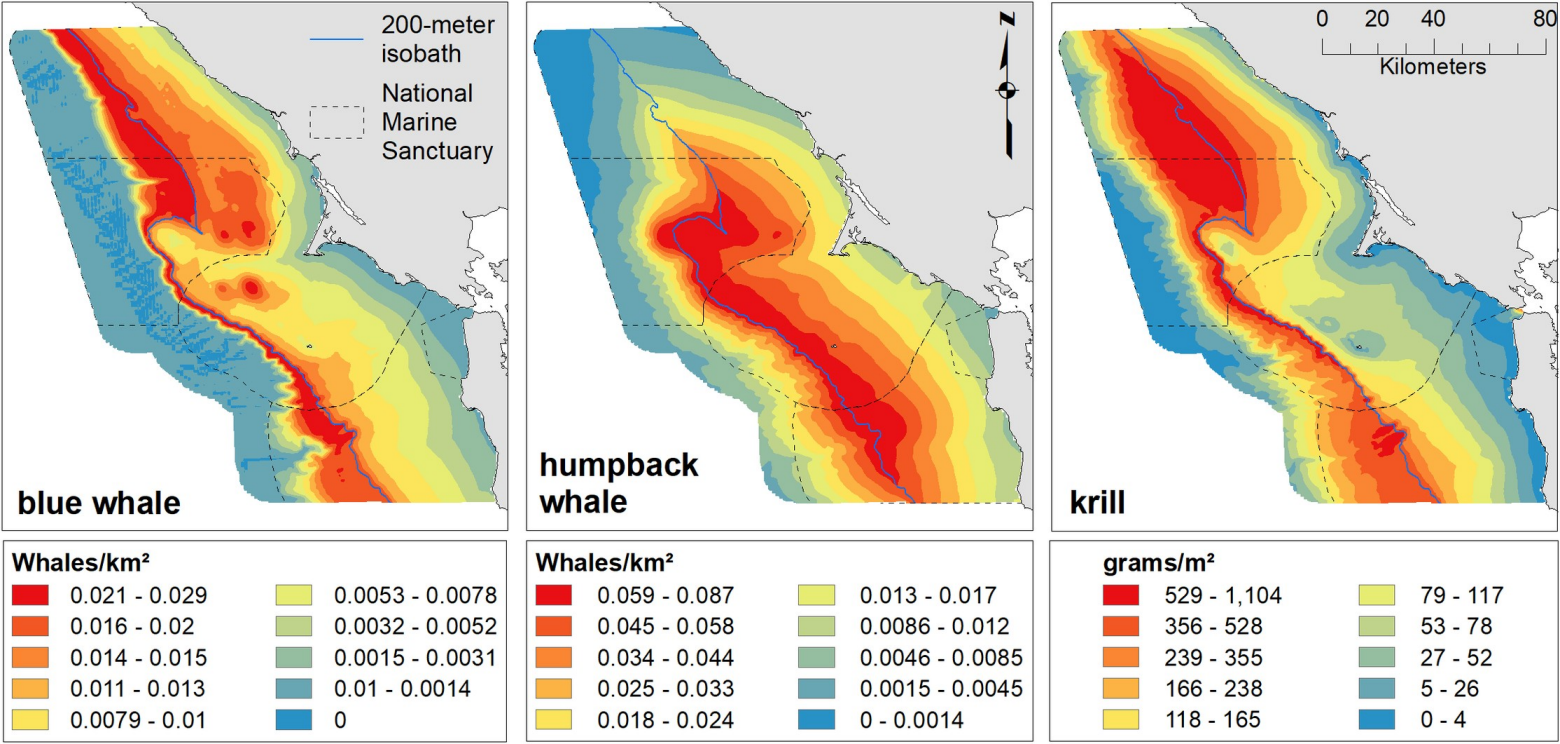
Mean predicted abundance of blue whale, humpback whale and krill. Predicted blue and humpback whale abundance and krill biomass, averaged across all months and years; Legend as in Fig 2.

Locations of high krill density varied across months (Fig 2) and years (Fig 3) for the 14-year study period. June and July exhibited the greatest amount of predicted krill with the highest values found along the 200 m isobath and north of Cordell Bank. Spatial patterns were similar across months, but krill extended further onto the shelf in July and especially June. The greatest amount of predicted krill occurred in 2017, with maximum values along the 200 m isobath. Two-thousand and nine, 2010, 2015 and 2016 were also notable for high krill abundance, while 2010 and 2016 had the broadest distribution of krill across the shelf. Two-thousand and seven and 2013 were low krill years with densities largely restricted to just offshore from the 200 m isobaths. In most years, krill were associated with the shelf break, though the strength of that relationship varied with year (Fig 3). Across all months and years, krill hotspots corresponded with the shelf break; there was high concentrations around the 200 m isobath and north of Cordell Bank (Fig 4).

### Blue whale habitat association and predicted distribution

Three oceanographic variables significantly predicted blue whale abundance (S2 and S4 Tables): surface temperature, surface fluorescence and midwater salinity. In addition, distance to the 200 m isobaths, depth, 3-month lagged UI and SOI, and PDO remained in the final model. An interaction between year and 200 m isobath also resulted in a lower AIC score.

**Fig 5:**
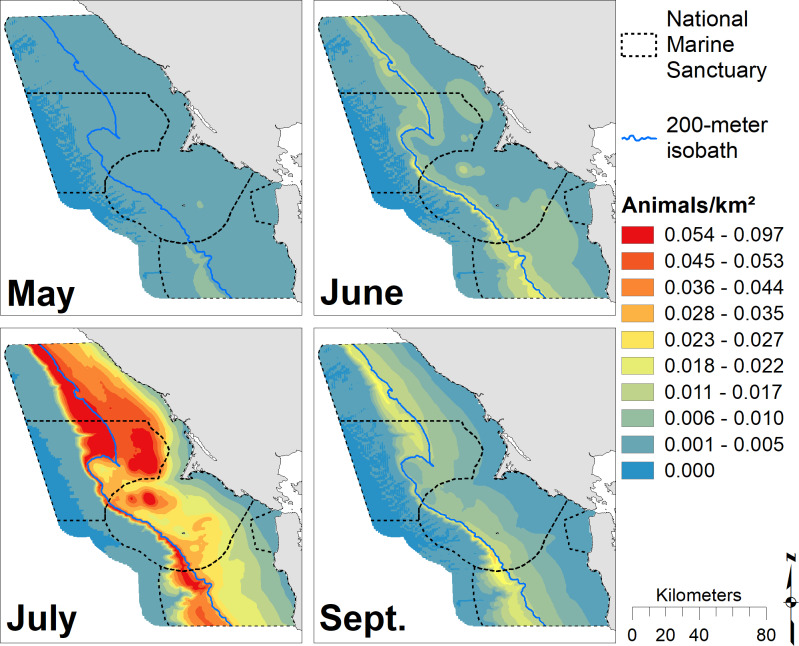
Modeled monthly blue whale abundance. Predicted blue whale abundance for May (predictions averaged over n = 8 years), June (n = 7), July (n = 7), and September (n = 8); Legend as in Fig 2.

**Fig 6:**
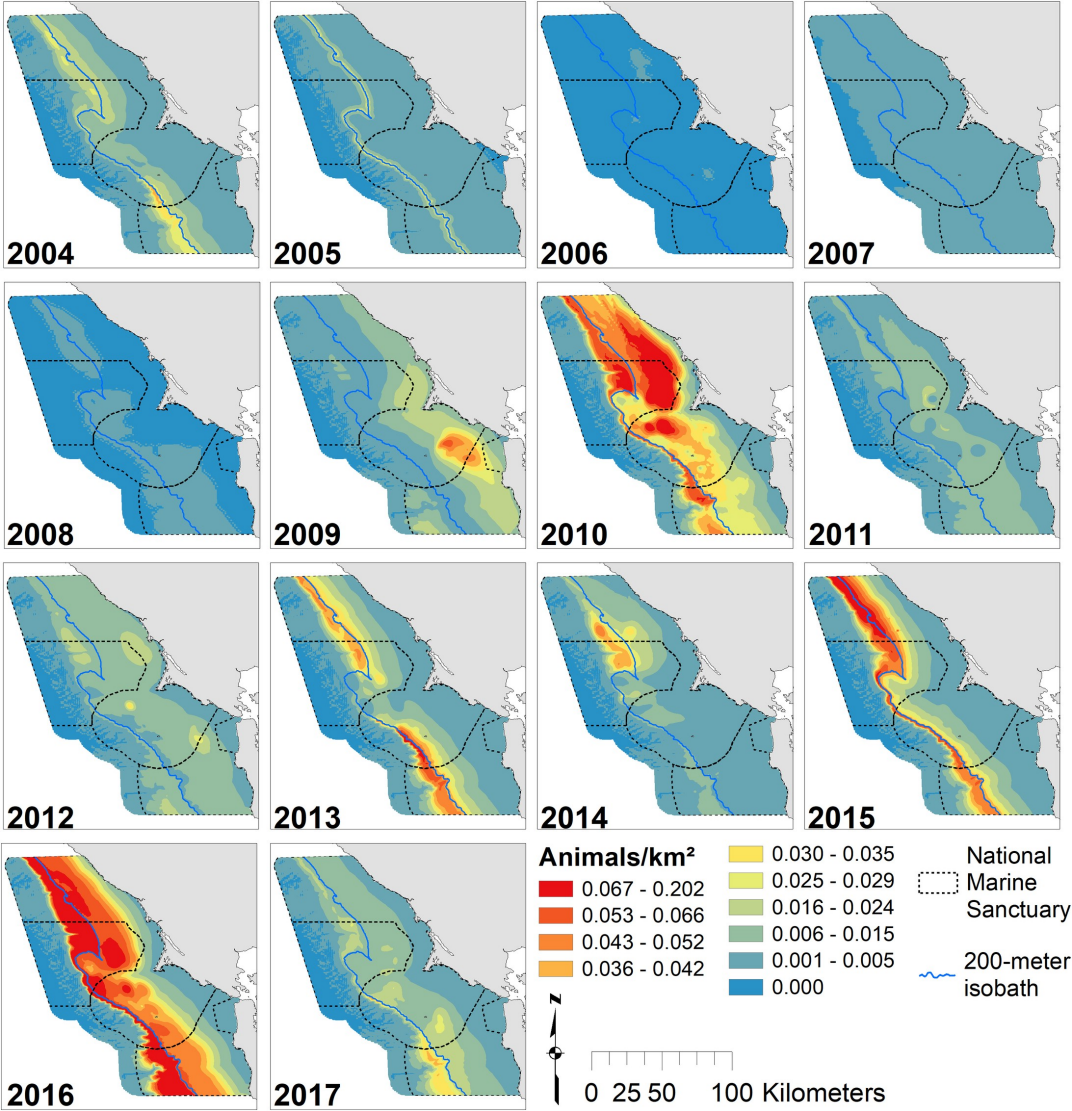
Modeled annual blue whale abundance. Predicted blue whale abundance, 2004–2017: variations in annual modeled habitat use (predictions averaged over n = 3 months except 2010, n = 4; 2013–14 and 2016–17, n = 2); Legend as in Fig 2.

Mean blue whale abundance had the highest values in July (Fig 5), though the model fit predicts peak abundance occurs in the un-sampled month of August (S4 Fig). Abundance varied more strongly across years than krill or humpback abundance (Fig 6). Moderate densities in 2004 were followed by several years of lower abundance until 2009. The highest predicted abundances were in 2010, 2015 and 2016. In 2010 and 2016, higher overall abundances resulted when blue whales were more broadly distributed across the shelf, while in 2015, they were strongly associated with the shelf break. In most years, the peak abundances occurred at the 200 m isobath or within 10 km. Two thousand and seven, 2009 and 2012 were exceptions, with peak density reaching a distance of 30 km in 2009 (S5 Fig). However, on average, blue whales had high densities centered around the 200 m isobaths and extending onto the shelf, with the exception of north of Cordell Bank where higher abundances were predicted offshore (Fig 4). Over the study period, blue whales were about 1.8 to 2.5 times more abundant in the vicinity of Cordell Bank than in areas around Southeast Farallon Island (Fig 4).

The blue whale model accurately predicted seasonal and spatial abundance patterns when compared to patterns of ACCESS cruise sightings. For example, in May, predictions were very low (Fig 5), and there were only 9 whales seen during ACCESS cruises over the 14 years compared to 158 seen during June cruises. K-fold cross validation showed good predictive capability with RMSE and MAE of the training and test sets matching closely (Table 2). Visual comparison of model predictions with sightings showed good concurrence in spatial distributions across months.

**Table 2:** (a) Species model results with transformation (L = linear, Q = quadratic, C = cubic, Qu = quartic) and coefficient sign for included model variables. Predictive models used a two-part model combining logistic and negative binomial regressions for krill (*n* = 2991) and a negative binomial regression for blue and humpback whales. The bold variable represents interaction of that variable with year. *P*-values: ‡ < 0.06; * < 0.05; ** < 0.01; *** < 0.0001. Model assessment metrics for goodness of fit and predictive ability are also reported. RMSE = root mean squared error, MAE = mean absolute error. Climate indices are abbreviated as in Table 1.

Variable	Krill: part 1 (zero)	Krill: part 2 (count)	Blue Whale	Humpback Whale
Temperature, surface			Q (-) ***	Q (-) ***
Temperature, midwater	Q (+) ***			Q (+) ***
Salinity, surface				C (+) **
Salinity, midwater	L (-) *	L (+) **	C (+) ***	Q (-) ***
Fluorescence, surface		Q (+) ***	C (-)‡	C (-) **
Fluorescence, midwater	L (+) ***	Q (+) ***		Qu (-) **
Distance to mainland	Q (-) **	Q (-) ***		
Distance to island	Q (+) *	Q (-) ***		Q (-) ***
Distance to Cordell		Q (-) *		
Distance to 200 m isobath	Q (+) *		Q(-)**	L (-) ***
Average depth		Q (-) ***	Q (-) **	L (-) **
Contour index	Q (+) **	L (+) ***		
SOI	Q (+) ***	Q (+) ***	L (+) ***, 3 month lag	L (+) **, 3 month lag
PDO	Q (-) ***	Q (-) *	L (-) *	L (-) *, 2 month lag
NPGO	Q (-) ***	Q (-) ***		L (-) *, 1 month lag
UI value	Q (-) ***, 3 month lag	Q (+) ***, 1 month lag	L (-) ***, 3 month lag	L (-) ***, 3 month lag
**Model Assessment Metrics**				
Pseudo-R^2^ (Nagelkerke)	-		0.237	0.160
RMSE (training)	1919.9		0.433	1.519
RMSE (k-fold CV)	1993.3		0.439	1.523
MAE (training)	756.0		0.113	0.651
MAE (k-fold CV)	775.4		0.109	0.621
Null Deviance	-		1007.46	2794.8
Residual Deviance	-		495.48	2002.7
Model Pr(>Chi)	< 0.0001		< 0.0001	< 0.0001

### Humpback whale habitat association and predicted distribution

Humpback whale habitat use was associated with both midwater and surface measures of all three oceanographic variables: fluorescence, temperature and salinity. In addition, depth, distance to the shelf break, distance to islands and four climate indices (UI, PDO, NPGO and SOI) also remained as significant covariates in the final model (S2 and S5 Tables). Humpback whale predictors included all the variables selected for blue whales and had similar response shapes with a few exceptions (Table 2 and S4 and S6 Figs). Humpback whales were associated with slightly warmer surface temperatures, more moderate midwater salinity and shallower depths than blue whales (S6 Fig).

**Fig 7:**
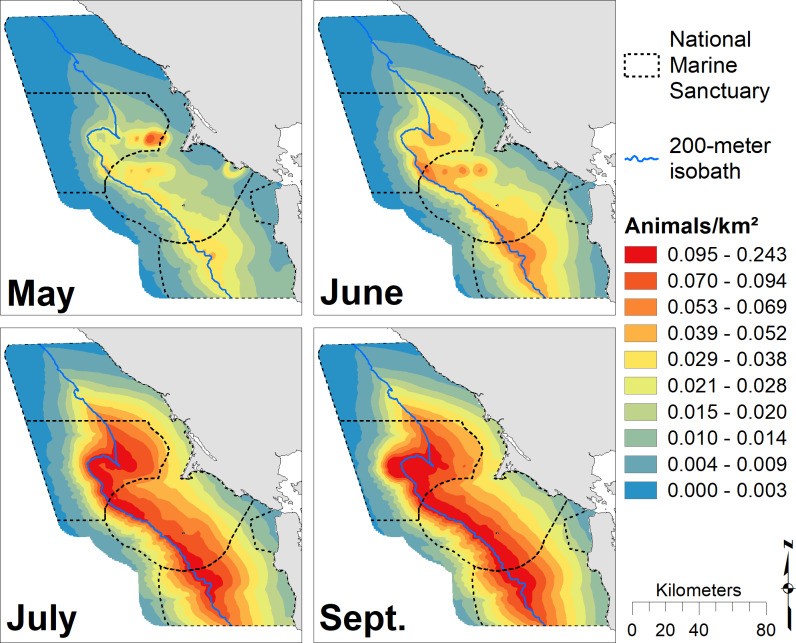
Modeled monthly humpback whale abundance. Predicted humpback whale abundance for May (predictions averaged over n = 8 years), June (n = 7), July (n = 7), and September (n = 8); Legend as in Fig 2.

**Fig 8:**
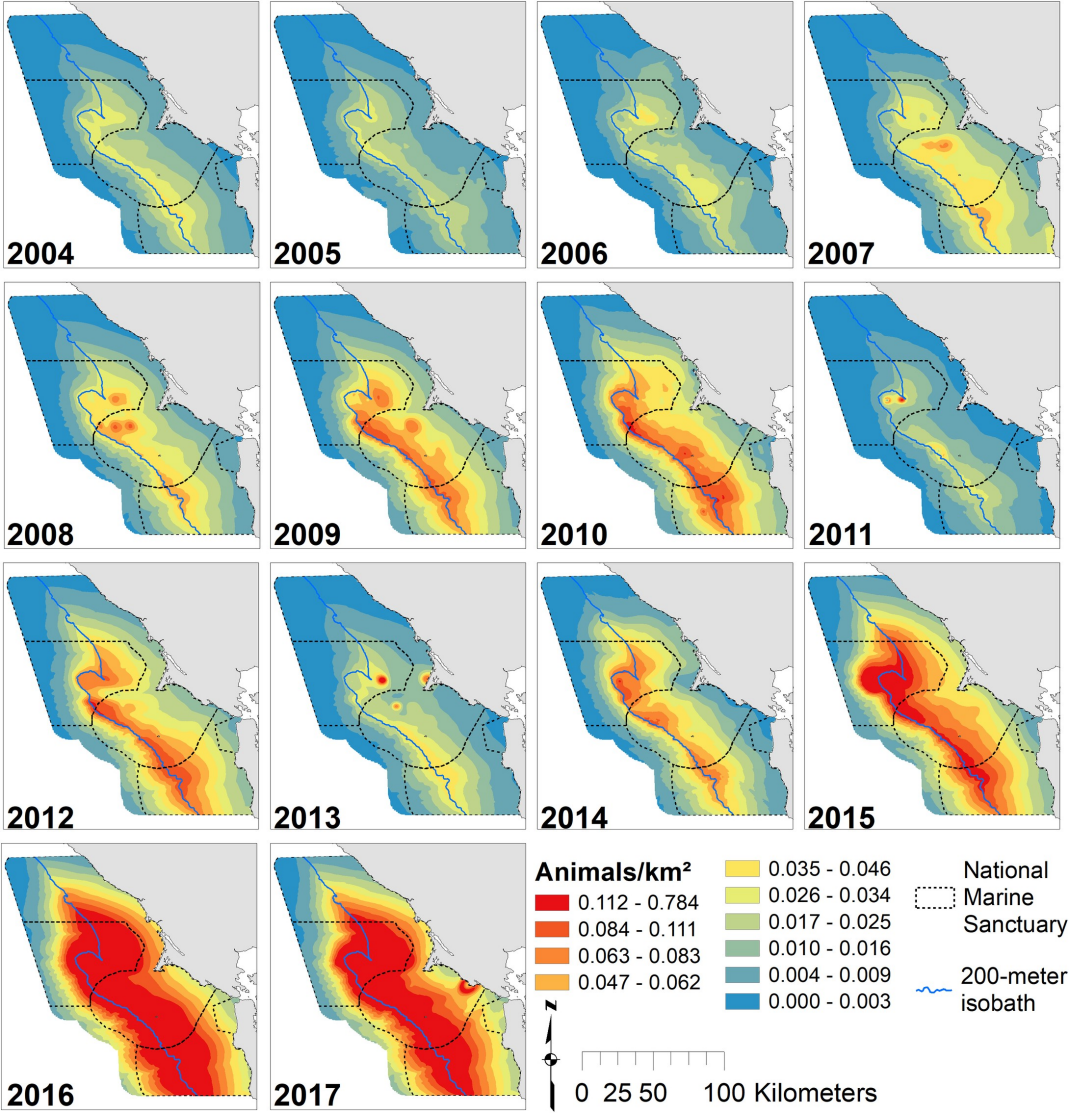
Modeled annual humpback whale abundance. Predicted humpback whale abundance, 2004–2017: variations in annual modeled habitat use (predictions averaged over n = 3 months except 2010, n = 4; 2013–14 and 2016–17, n = 2); Legend as in Fig 2.

Humpback whale predicted density peaked in September, and whales became progressively more broadly distributed over the season from May through September (Figs 7 and S6). May density was low and patchy, but highest values were inshore from Cordell Bank. Overall, humpback whales were strongly associated with the shelf break, but showed high predicted abundances over significant regions of the shelf (Fig 4). The largest region of high-abundance was over Cordell Bank and both to the west and east. Our models predicted lower abundance years early in the study (2004–2006) and in 2011 and 2013. Peak predicted humpback whale abundance was in 2017 (the highest predictions), 2016 (the broadest high abundances) and 2015 (Fig 8). Similar to blue whales, lower abundance years tended to have patchier distributions, but usually also included Cordell Bank as one of the highest abundance areas.

### Krill and whale hotspots

**Fig 9:**
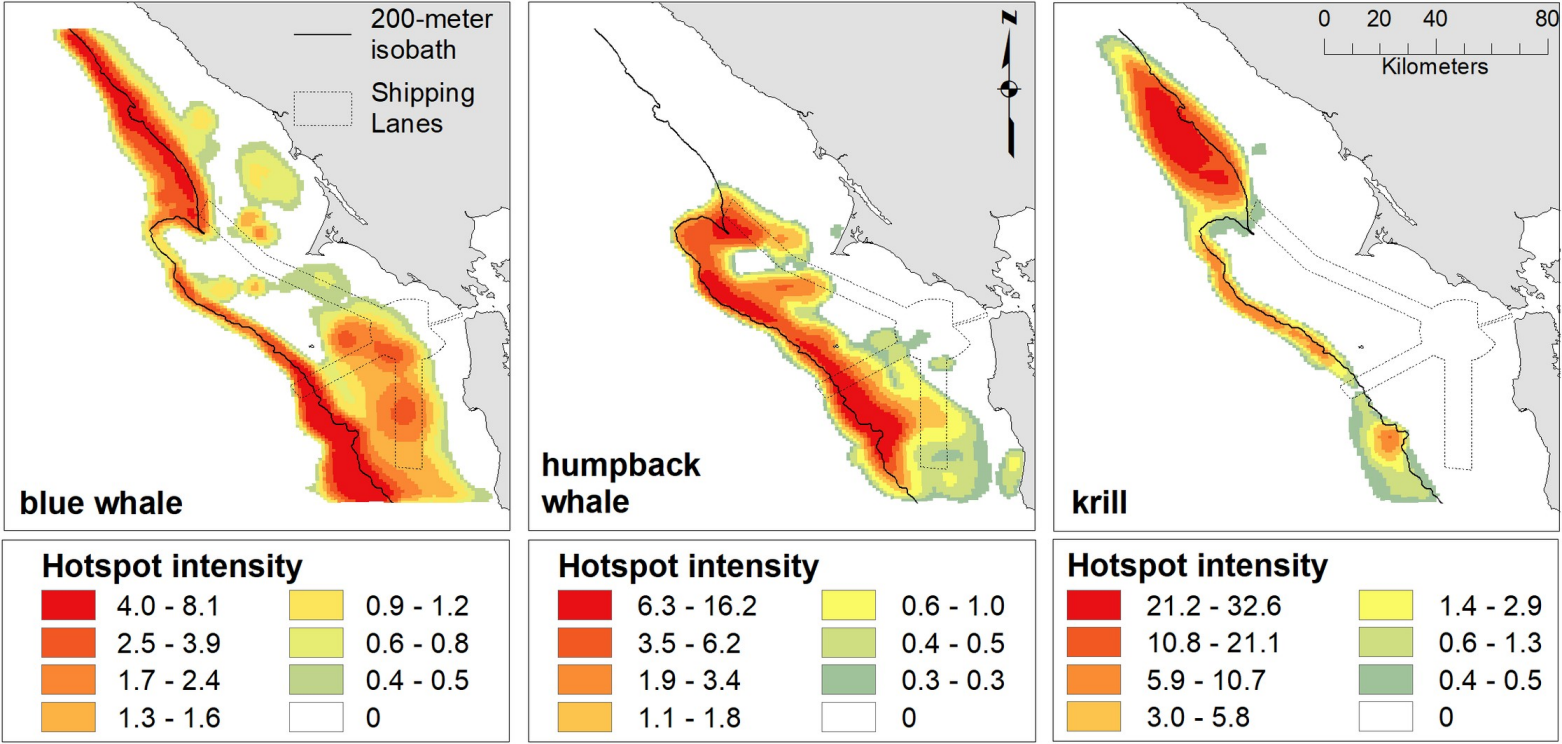
Hotspot intensity for blue whales, humpback whales and krill. Hotspots are the sum of normalized (0–1) Getis-Ord G_i_* significant positive z-scores for modeled abundances from cruises during May—July and September of 2004–2013. The 200 m isobath and boundaries of the Traffic Separation Scheme are included in solid and dashed black, respectively.

Hotspot analysis using the Getis-Ord G_i_* statistic showed overlapping, but distinct patterns of high spatial use for the two whale species but much stronger concurrence between blue whales and krill than humpback whales and krill (Fig 9). Blue whales displayed strong hotspots along the shelf break, especially to the north of Cordell Bank and south of the Farallon Islands. Additional, lower value hotspots occurred on the shelf off of Bodega Bay and in a region inshore and south of the Farallon Islands. In contrast, humpback whale hotspots were largely from Cordell Bank south and extended onto the shelf inshore of the shelf break. Krill hotspots were strongest north of Cordell Bank and offshore of the 200 m isobaths but also extended along the shelf break to the south.

### Trophic co-occurrence of whales and krill

**Fig 10:**
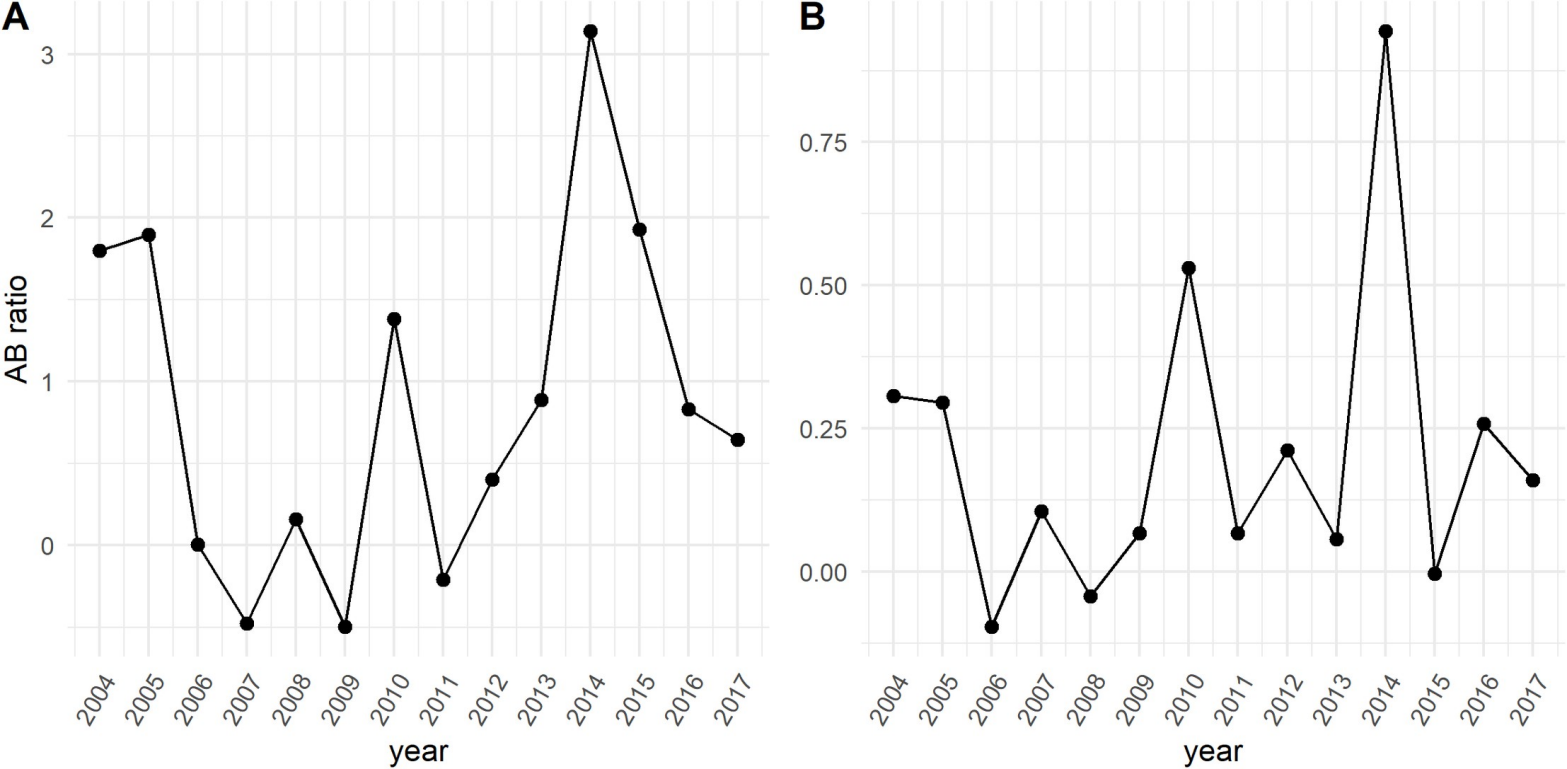
Annual AB ratio calculated for blue (A) and humpback (B) whale overlap with krill. Positive ratios indicate strong overlap, zero is no pattern of overlap and negative values can indicate different habitat use between species or avoidance of predators by the prey.

We examined annual variation in trophic overlap (AB ratio) between each whale species and krill (Fig 10). Significant variability occurred between years for both blue and humpback whales and in each case, three years showed negative AB ratio values, though the years were different. Overlap was low or negative from 2006 to 2009 for both species. In addition, blue whales had a negative ratio in 2011, while humpback whale AB ratio was negative in 2015. Overall, blue whales had much greater AB ratios than humpback whales.

**Fig 11:**
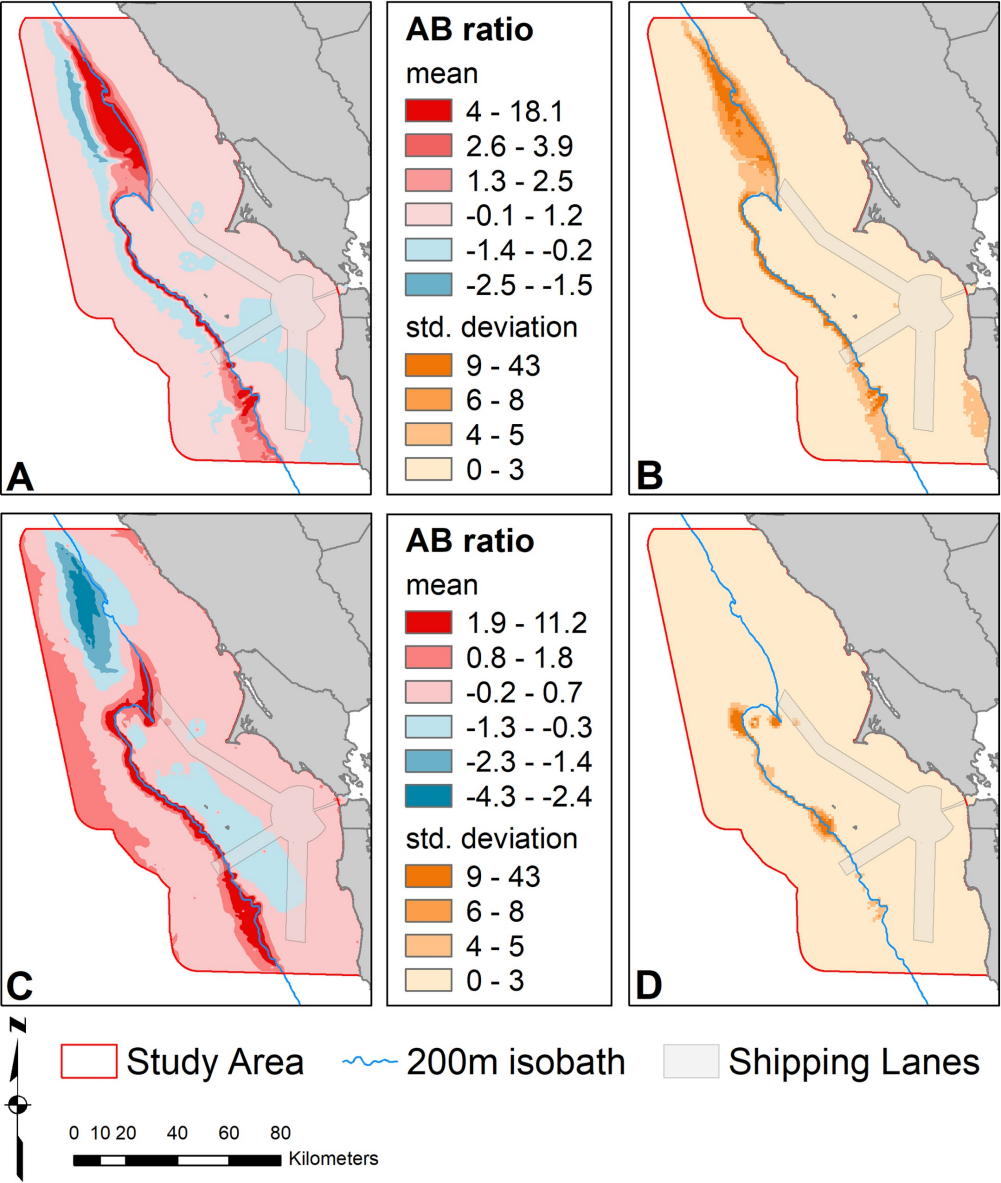
Mean (A, C) and standard deviation (B, D) of AB ratio calculated across all modeled months for blue (A, B) and humpback (C, D) whale overlap with krill. Positive mean ratios indicate overlap, zero is no overlap and negative values can indicate different habitat use between species, or avoidance of predators by the prey. Higher standard deviation indicates greater variation in degree of whale overlap with krill across months.

The time-averaged, spatially explicit AB ratios showed strong trophic overlap of blue whales with krill in the area off the shelf and north of Cordell Bank (Fig 11). In contrast, this area had negative AB ratios for humpback whales, indicating krill there are not heavily targeted by this species. Both species show elevated AB ratios along the shelf break the south of Cordell Bank, but humpback whales had higher trophic overlap with krill immediately north, east and over Cordell Bank itself. In addition, high standard deviation of AB ratios are found over a broader area for blue whales compared to humpback whales. While both species are targeting and utilizing krill along the shelf break, there is some separation in space. Humpback whale trophic overlap is more persistent in time, but lower than that of blue whales.

## Discussion

### Krill habitat use

Krill hotspots within the California Current System consistently correspond with the shelf-break region, particularly waters around and north of Cordell Bank. Our models show the greatest predicted krill abundance in late spring and early summer. In the later years of our study, elevated krill densities corresponded with increased upwelling and more productive ocean conditions [70]. Krill are abundant in regions of increased upwelling and tend to aggregate near or along ocean-floor features like the steep sides of submarine canyons, subsurface plateaus, islands, or the shelf break [17,19,22,71].

Krill abundance was associated with climate indices and UI. In our modeling process, we included the possibility of up to a 3-month lag in each climate variable to allow for a delayed effect on prey and predators. However, with the exception of UI (which best represents local conditions), no time-lags on final model climate indices related significantly to krill abundance. Previous studies on euphausiids within the California Current System have found significant associations between krill and UI at 30-, 90-, or 365-day lags, demonstrating the dependence of adult krill survival and development on both short- and long-term ocean conditions [72,73].

The high productivity of the California Current sustains the krill in this region, which is specifically known for its upwelling intensity. The positive relationships between krill abundance and midwater salinity, cooler midwater temperature and high surface fluorescence, capture the increase in krill production that occurs in stratified, mature upwelled waters with abundant primary producers [16]. In non-productive years (2004–05, 2007 and 2013), our models predicted krill density to be highest close to the shelf break. In productive years (2008–11 and 2015–17), krill were predicted to be more broadly distributed over the continental shelf. However, adjacent and upstream areas are also critical to the krill life cycle, as advection by ocean currents may be a first-order control on the health and abundance of krill individuals in northern California waters [74].

Our model showed a strong association between krill and bathymetric-related variables over the course of the local upwelling period as well as in early fall. Of those, one of the strongest was the shelf break. These results agree with a recent modeling study off central and southern California, where the greatest concentrations of krill were found along the shelf break [16]. Similarly, krill density assessed from independent surveys also found high abundances along the shelf and near Cordell Bank, though variability in hotspot formation was greater at Cordell Bank compared to south along the shelf [75].

### Blue whale habitat use

Our models confirm previous findings: blue whales prefer habitat near the shelf break and aggregating bathymetric features that lead to accumulation and retention of krill [31,34,65,76]. We found areas of high blue whale concentration on the outer edge of the continental shelf, particularly areas east and north of Cordell Bank. Predicted whale abundance was highest in late summer and fall, with the greatest predicted values in July. Similar to krill, whales were found in greater abundances in the second half of our study, a period that exhibited increased upwelling and more productive ocean conditions [70].

Blue whale abundance was associated with climate indices (PDO and SOI), bathymetry and local oceanography (UI, temperature, salinity, and fluorescence). Several other studies have shown temperature as the most significant variable in predicting blue whale habitat at various spatial scales [77–79], and our models showed a similar temperature relationship. Despite the relatively small area covered by our data, basin-scale indices also had good explanatory power for blue whale abundance patterns in our region. Recent work has highlighted the apparent capability of blue whales to sense ocean conditions at a variety of scales [80]. In addition, both long-term memory and response to dynamic conditions influence the use of persistent prey hotspots such as Cordell Bank [81].

Overall, blue whale abundance varied greatly among non-productive (2004–07) and productive (2008–11) conditions, with whales nearly absent in non-productive years. Benson *et al*. [26] found blue whales absent from Monterey Bay in years with El Niño-like conditions concomitant with a reduction in offshore zooplankton biomass. Lagged PDO and NPGO were significant in predicting whale abundance, and may be related to the timing of peak primary productivity as it moves north along the California coast. Some individuals consistently target persistent foraging areas such as the greater Farallones region, while others quickly leave in search of better foraging opportunities [8,81].

Bathymetric-related variables were also strongly associated with whale distribution in our model. This relationship likely emerges because bottom features create currents and convergences which aggregate krill. Since whales depend on krill patches with high local density, these features provide reliable foraging resources [18,65,76,82–84]. Other local studies of blue whales have shown that areas of steep bathymetry are associated with blue whale foraging in particular. For example, two whales tagged in Monterey Bay targeted prey and frequently dove to depths between 150 and 200 m, focusing foraging energy along the Monterey Submarine Canyon [65]. Schoenherr [18] also showed a close relationship between blue whales, euphausiids, and the Monterey Submarine Canyon’s edge. Prey in nearby Monterrey Bay are also aggregated along steep areas such as shelf breaks, which, coupled with highly productive waters, attract these predators [17,19,22].

### Humpback whale habitat use

Compared to blue whales, our model of humpback habitat use included many more local oceanographic predictors: both surface and midwater measures of all three local ocean variables remained in the model, suggesting that humpback whales rely more heavily on the immediate ocean conditions to guide habitat use. This discrepancy may also be indicative of the different feeding ecology of blue and humpback whales. Blue whales eat krill almost exclusively while humpback whales are generalist feeders, also consuming forage fish such as anchovy [81,85]. Since forage fish are highly mobile, their distribution may mirror contemporaneous oceanography more closely than less mobile krill. For variables in both species models, the functional shapes of predictors were similar for humpback and blue whales (i.e., the same transformation was selected) (S4 and S6 Figs). One exception was distance to the shelf break which decreased linearly for humpbacks compared to the quadratic relationship of blue whales. This difference matches a known difference in ecology between the species: humpback whales are more broadly distributed across the shelf and occur further inshore [34].

### Hotspots, spatial co-occurrence and management implications

Our models of blue and humpback whales support multiple studies that have identified habitat and resource partitioning between sympatric whale species [33,34,86–89]. Similar to the findings of Fossette *et al*. [34], our results show that blue whales are more strongly associated with the shelf break (and the dense krill there), while humpback whales are more broadly distributed on the shelf likely due to their ability to switch between krill and fish prey [34,85]. In addition, our modeled peak of blue whale abundance coincides with that for krill (August), while humpback whale abundance peaks later in the season (September/October), after krill abundance has begun to decline (Figs 2, 5 and 7). Despite their greater flexibility in diet, humpback whales are known to extensively utilize krill and may target peak krill production in our region during migration [34]. However, the better alignment of blue whale abundance with krill makes sense given that they are exclusively krill predators [81]. The stronger association of humpback whales with the shelf break in May and June suggests they target krill early in the season. Later in the season (July and September), a broader distribution across the shelf break (Fig 7) supports multiple lines of evidence that humpback whales switch to consuming more forage fish as krill productivity decreases [34,85].

Our model results show good evidence for temporal niche separation and some support for spatial segregation between blue and humpback whales. Our hotspot and trophic overlap analysis provides additional verification of resource partitioning. While blue and humpback whale hotspots overlap, the highest hotspot areas for krill coincide only with blue whale hotspots (Fig 9). The higher trophic overlap offshore for blue whales coincides with their greater ability to utilize deeper prey (Fig 11A). Humpbacks, on the other hand, overlap more in shallower waters and where Cordell Bank aggregates krill at shallower strata (Fig 11C).

Due to their size, blue whales have higher feeding rates than other baleen whale species and require high density krill patches to achieve positive energetic intake [35,36,90]. For example, Goldbogen *et al*. [91] found blue whales dove more than twice as frequently as bowhead whales to similar foraging depths. Our krill model only predicts average density across space and time, rather than occurrence of the high-density patches required by blue whales. This difference may explain why blue whales show distributions and hotspots further inshore than krill (Figs 4 and 9) that don’t align with the trophic overlap pattern (Fig 11). When krill patches occur both on and off the shelf, it is more energetically efficient to dive in shallower waters if patches of krill are of similar density [90]. In addition, during periods with low overall krill abundance and productivity, patchy but dense krill aggregations may serve as important resources for blue whales.

Hotspots and high trophic co-occurrence for both blue and humpback whales occur at the end of the northern shipping lane (Figs 9 and 11). Large whales are vulnerable to ship strikes, especially in areas where high numbers of vessels are traveling quickly [92–94]. Some research suggests blue whale populations have not rebounded to pre-whaling numbers in part due to ship strikes in high vessel-traffic areas [8,94–96] (although see [97]). The incidence of strikes is most likely underreported [94,96,98,99]; since whales are negatively buoyant, they generally sink rather than float or strand ashore [100] where they would be more easily observed. Thus, the true mortality rate is likely more than 10 times higher than the reported number of stranded carcasses [94,99]. Within the Sanctuaries, there have been 20 reported ship-strike mortalities and at least 10 injured or possibly killed whales of varied species between 1988 and 2011 [101]. In 2010 alone, there were 5 fatalities, one a near-term pregnant blue whale (J. Jahncke, personal observation).

In areas of high use by both humans and threatened species, limited management has been implemented to reduce possible ship strike incidents, although the effectiveness of these attempts, especially within CBNMS and GFNMS is in doubt [102,103]. In 2013, the overall footprint of San Francisco Bay traffic was modified to improve maritime safety. However, while this action decreased the area of human use in these sanctuaries, it concentrated traffic through the major whale hotspot north and east of Cordell Bank, highlighted in this paper. Since 2014, the sanctuaries have implemented a voluntary speed request during the months when whales are most abundant. However, cooperation with the program has been limited, and still falls well below what has been achieved in other regions where speed limits are mandatory [103]. Enforceable seasonal restrictions, rather than voluntary requests, have been successfully implemented to protect whales elsewhere (e.g., Stellwagen Bank National Marine Sanctuary). Implementing seasonal restrictions on vessel speed and route usage to avoid whale hotspots identified in this study can reduce the vulnerability of whales to ship strikes in Sanctuaries’ waters.

Fuel-usage recommendations and regulations are also likely impacting the risk of whale strikes in CBNMS and GFNMS. Fuel type requirements can change vessel traffic patterns by requiring different fuels and emissions levels near the coast. Effective as of June 2009, the California Air Resources Board Ocean-Going Vessel (OGV) Fuel Rule required vessels within 24 nmi of the California coastline to use more expensive low-sulfur fuels [101,102,104]. This ruling reduced potential vessel-interaction with blue whales when the majority of traffic in and out of San Francisco Bay shifted routes from use of southern and northern lanes to western lanes, away from the coast, in attempts to reduce time spent using more expensive low-sulfur fuels [105]. However, in early 2015, a different federal regulation took precedence over the California Air Resources Board, requiring vessels within 200 nmi of the United States to use fuels with an oil sulphur limit of 0.1% m/m to achieve lower particulate matter emissions [104]. With this new regulation, the financial incentive to stay off the coast disappeared. Since this change, traffic has shifted back to using the north and south lanes, likely increasing risk to whales [104].

We encourage managers to utilize our identified hotspots to protect foraging whales by implementing a seasonal management area to slow vessel traffic and increase caution in these areas in late spring to early fall. One solution to this challenge would be to eliminate all but the western lane, focusing traffic back to the route that most avoids whale density and important feeding habitat (Fig 11). This action, in addition to requiring seasonal speed and route changes, will help mitigate the number of potential lethal encounters ships have with foraging whales within the Sanctuaries.

## Supporting information

S1 File Description of methods for calculating g(0) and ESW and partial plots of model covariates for krill, blue whale and humpback whale models. (DOCX)

S1 Fig (DOCX)

S2 Fig (DOCX)

S3 Fig (DOCX)

S4 Fig (DOCX)

S5 Fig (DOCX)

S6 Fig (DOCX)

S1 Table (DOCX)

S2 Table (DOCX)

S3 Table (DOCX)

S4 Table (DOCX)

S5 Table (DOCX)
