# Supplementary material for: Modeling predator and prey hotspots: Management implications of baleen whale co-occurrence with krill in Central California
Source: PLoS One. 2020 Jul 7;15(7):e0235603. doi: 10.1371/journal.pone.0235603 (PMC7340285; doi:10.1371/journal.pone.0235603)
Supplement: S1 File — (DOCX) [file pone.0235603.s001.docx]

­S1 Supporting Information

## Adjusting detection probability by modeling effective strip width using survey covariates

To more accurately account for variation in survey effort, we used multi-covariate distance sampling (MCDS) and the R package ‘mrds’ (Laake, Borchers, Thomas, Miller, & Bishop, 2015) to estimate the effective strip width (ESW) for each track line bin. MCDS accounts for variation in detection probability that occurs as a result of environmental conditions such as fog or sea state during the survey (Buckland et al., 2004). Previous work by Barlow *et al.* (2001) found that pooling species into categories with similar sighting characteristics improved model performance when analyzing distance sampling data by increasing sample sizes without losing information. We therefore pooled sighting data from ACCESS for ‘large whale’ species (including all mysticetes plus killer whales). Covariates considered for model inclusion were: Beaufort Sea State (*Beaufort*, ordinal 0-6), swell height (*SwellHt*, continuous estimate in meters), visibility (*Vis*, ordinal estimate of distance at which observers could detect cetaceans), and weather (*Weather*, categorical variable for haze, fog, rain or fog and rain). Because the presence of fog in the ACCESS survey region is common and significantly affects visibility in the field, we also simplified *Weather* into a binary variable, *Fog*, for the presence or absence of fog.

Perpendicular distance to whale sightings was only recorded for surveys after 2009. We therefore used this more recent data to fit and select MCDS models. The selected models were then also used to predict ESW for surveys from 2004-2009 under the assumption that the shape of the detection function and its relationship with environmental covariates remained the same across years. We used forward stepwise model building and a hazard-rate key function (Buckland et al., 2004). Starting with the most significant predictor variable for both species groups, *Fog*, we added the variable to the model that provided the lowest Akaike Information Criterion (AIC) until all candidate variables were used. The models within +/- 2 AIC units of the lowest AIC model were used to predict ESW (Table 1).

We also used our data set to calculate Beaufort-specific g(0) values for blue whales following Barlow *et al.* (2015)(Table 2). g(0) represents the probability of detecting a whale along the track-line and is often assumed to be 1 (100% detection) for all conditions. The methods of Barlow and colleagues allow estimation of decreased detection as sighting conditions deteriorate with increasing sea state. Our calculated g(0) values allow us to correct for this detection bias. ESW, bin length and g(0) value were multiplied for each bin resulting in an effort (effectively surveyed area) and detection bias correction factor. We used this value as an offset in the modeling of blue whale counts.

**References**

Barlow, J. (2015). Inferring trackline detection probabilities, g(0), for cetaceans from apparent densities in different survey conditions. *Marine Mammal Science*, (0), 1–21. http://doi.org/10.1111/mms.12205

Barlow, J., Gerrodette, T., & Forcada, J. (2001). Factors affecting perpendicular sighting distances on shipboard line-transect surveys for cetaceans. *Journal CETACEAN RES. MANAGE*, *3*(2), 201–212.

Buckland, S. T., Anderson, D., Burnham, K., Laake, J., Borchers, D., & Thomas, L. (2004). *Advanced distance sampling*. New York: Oxford University Press.

Laake, J., Borchers, D., Thomas, L., Miller, D., & Bishop, J. (2015). mrds: Mark-Recapture Distance Sampling.
