# Supplementary material for: Modeling predator and prey hotspots: Management implications of baleen whale co-occurrence with krill in Central California
Source: PLoS One. 2020 Jul 7;15(7):e0235603. doi: 10.1371/journal.pone.0235603 (PMC7340285; doi:10.1371/journal.pone.0235603)
Supplement: S3 Fig — (DOCX) [file pone.0235603.s004.docx]

**Figure 3.** Marginal effect of contour index conditioned on year for krill mass. The marginal change to predicted krill mass is plotted on the y-axis. Points represent the partial residuals of each observation and the blue shaded area is the 95% confidence interval. The marginal effect is calculated by holding all other variables at their mean value.

**
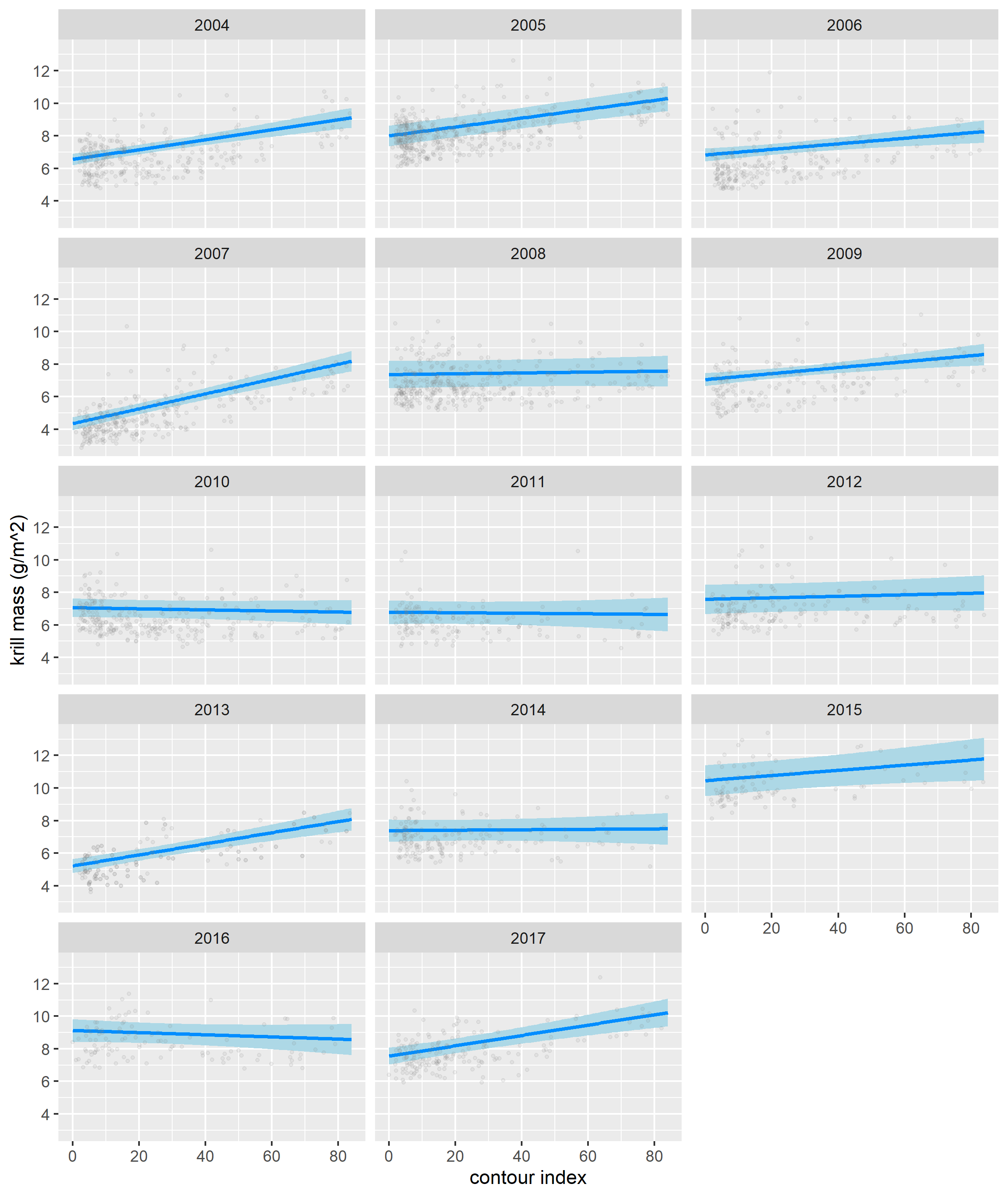
**
