# Supplementary material for: Modeling predator and prey hotspots: Management implications of baleen whale co-occurrence with krill in Central California
Source: PLoS One. 2020 Jul 7;15(7):e0235603. doi: 10.1371/journal.pone.0235603 (PMC7340285; doi:10.1371/journal.pone.0235603)

**Figure 5.** Marginal effect of distance to the 200m isobaths (shelf break) conditioned on year for blue whale counts. The marginal change to predicted blue whale abundance is plotted on the y-axis. Points represent the partial residuals of each observation and the blue shaded area is the 95% confidence interval. The marginal effect is calculated by holding all other variables at their mean value.


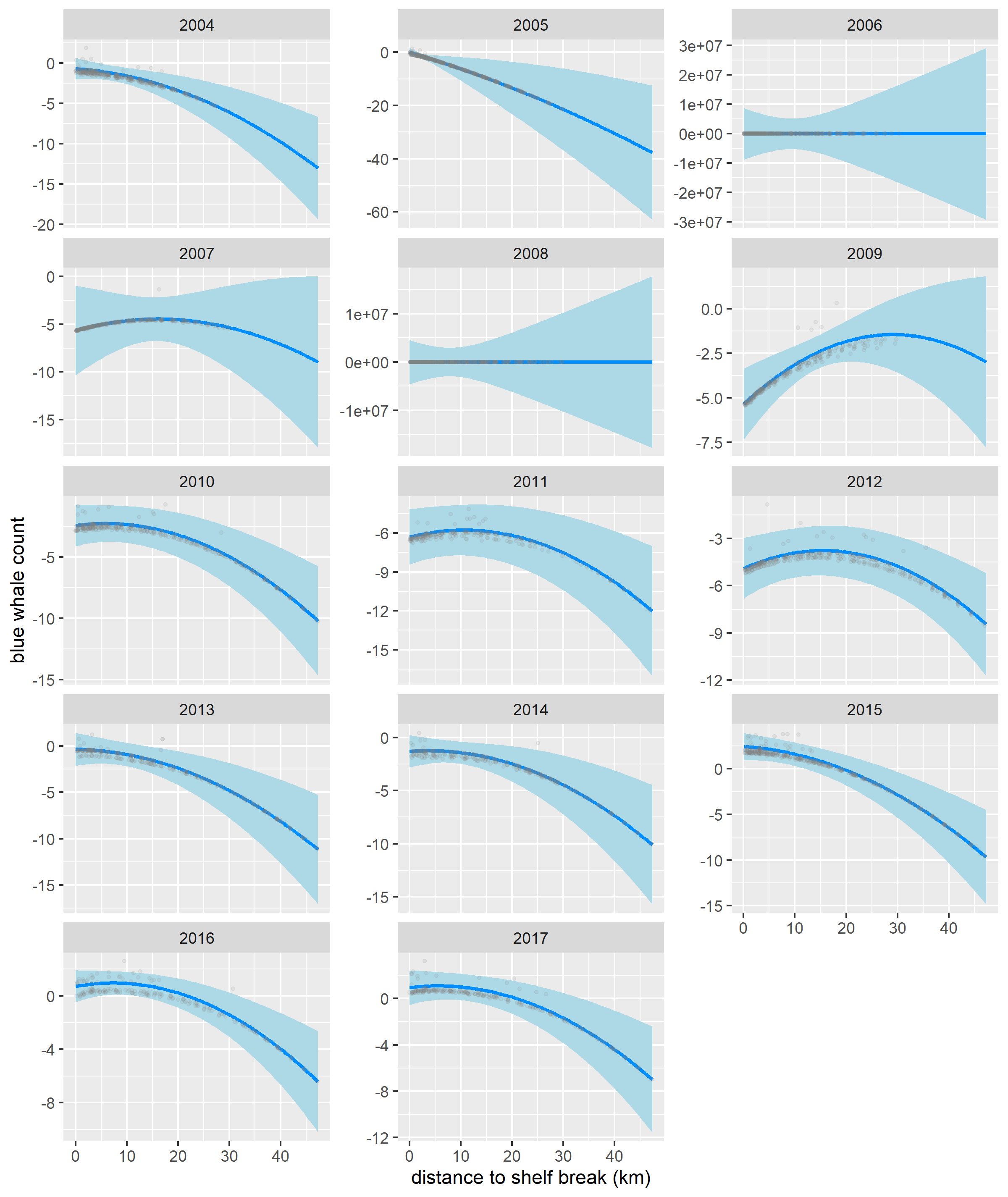

Supplement: S5 Fig — (DOCX) [file pone.0235603.s006.docx]
