# Supplementary material for: Modeling predator and prey hotspots: Management implications of baleen whale co-occurrence with krill in Central California
Source: PLoS One. 2020 Jul 7;15(7):e0235603. doi: 10.1371/journal.pone.0235603 (PMC7340285; doi:10.1371/journal.pone.0235603)
Supplement: S1 Table — (DOCX) [file pone.0235603.s008.docx]

**Table 1**. Average multiplicative effect of Beaufort on blue and humpback whale ESW for all track segments from 2004-2017 with all other model covariates held constant at their mean values.

|  | Average effect | |
| --- | --- | --- |
| Beaufort | Blue whale | Humpback whale |
| 0 | 1.2955 | 1.1012 |
| 1 | 1.1596 | 1.0440 |
| 2 | 1.0355 | 0.9893 |
| 3 | 0.9228 | 0.9371 |
| 4 | 0.8210 | 0.8873 |
| 5 | 0.7294 | 0.8398 |
| 6 | 0.6474 | 0.7946 |
