# Supplementary material for: Modeling predator and prey hotspots: Management implications of baleen whale co-occurrence with krill in Central California
Source: PLoS One. 2020 Jul 7;15(7):e0235603. doi: 10.1371/journal.pone.0235603 (PMC7340285; doi:10.1371/journal.pone.0235603)
Supplement: S2 Table — (DOCX) [file pone.0235603.s009.docx]

**Table 2**. Estimated probabilities of detecting blue and humpback whales along the survey track line during survey segments at sea state values from 0-6. Probability of detection at sea state 0 (calm conditions) is assumed to be perfect.

|  | g(0) estimate | |
| --- | --- | --- |
| Beaufort | Blue whale | Humpback whale |
| 0 | 1 | 1 |
| 1 | 0.9563 | 0.8559 |
| 2 | 0.9146 | 0.7326 |
| 3 | 0.8746 | 0.6270 |
| 4 | 0.8363 | 0.5367 |
| 5 | 0.7996 | 0.4594 |
| 6 | 0.7645 | 0.3931 |
