# Supplementary material for: Modeling predator and prey hotspots: Management implications of baleen whale co-occurrence with krill in Central California
Source: PLoS One. 2020 Jul 7;15(7):e0235603. doi: 10.1371/journal.pone.0235603 (PMC7340285; doi:10.1371/journal.pone.0235603)
Supplement: S3 Table — (DOCX) [file pone.0235603.s010.docx]

**Table 3.** Coefficients, standard errors, z values and p values for all quantitative variables (including year and interactions with year) for the two-part model combining logistic and negative binomial regression for krill.

| Count model coefficients (truncated negbin with log link): | | | | |  |
| --- | --- | --- | --- | --- | --- |
|  | **Estimate** | **Std. Error** | **z value** | **Pr(>\|z\|)** |  |
| (Intercept) | -34.180857 | 7.66611 | -4.459 | 0.000008 | *** |
| year (2005) | 1.534265 | 0.372063 | 4.124 | 0.000037 | *** |
| year (2006) | 0.274951 | 0.269325 | 1.021 | 0.307307 |  |
| year (2007) | -2.506931 | 0.373448 | -6.713 | 0.000000 | *** |
| year (2008) | 0.782388 | 0.676643 | 1.156 | 0.247568 |  |
| year (2009) | 0.493564 | 0.301245 | 1.638 | 0.101336 |  |
| year (2010) | 0.45494 | 0.434619 | 1.047 | 0.295213 |  |
| year (2011) | 0.158411 | 0.55326 | 0.286 | 0.774631 |  |
| year (2012) | 1.042184 | 0.67855 | 1.536 | 0.124563 |  |
| year (2013) | -1.466664 | 0.325711 | -4.503 | 0.000007 | *** |
| year (2014) | 0.891927 | 0.401205 | 2.223 | 0.026208 | * |
| year (2015) | 4.121275 | 0.537004 | 7.675 | 0.000000 | *** |
| year (2016) | 2.726788 | 0.42001 | 6.492 | 0.000000 | *** |
| year (2017) | 1.071105 | 0.293272 | 3.652 | 0.000260 | *** |
| contour index | 0.031159 | 0.004878 | 6.388 | 0.000000 | *** |
| month | 3.048736 | 0.30842 | 9.885 | < 2e-16 | *** |
| month² | -0.220943 | 0.021875 | -10.1 | < 2e-17 | *** |
| Surface Fluorescence | 3.334213 | 3.783157 | 0.881 | 0.378139 |  |
| Surface Fluorescence² | 9.259876 | 2.534466 | 3.654 | 0.000259 | *** |
| Midwater Fluorescence | 14.006559 | 3.789043 | 3.697 | 0.000219 | *** |
| Midwater Fluorescence² | 5.294945 | 3.37861 | 1.567 | 0.117069 |  |
| Midwater Salinity | 0.709112 | 0.231125 | 3.068 | 0.002154 | ** |
| distance to Cordell | 8.805041 | 3.479899 | 2.53 | 0.011398 | * |
| distance to Cordell² | -10.162629 | 4.080802 | -2.49 | 0.012762 | * |
| distance to island | 25.284978 | 3.86976 | 6.534 | 0.000000 | *** |
| distance to island² | -9.092592 | 3.106328 | -2.927 | 0.003421 | ** |
| distance to land | 9.83833 | 3.859458 | 2.549 | 0.010799 | * |
| distance to land² | -12.239946 | 3.079104 | -3.975 | 0.000070 | *** |
| average depth | 1.316585 | 3.033684 | 0.434 | 0.664297 |  |
| average depth² | -7.962381 | 2.371857 | -3.357 | 0.000788 | *** |
| SOI | -7.38397 | 4.985965 | -1.481 | 0.138620 |  |
| SOI² | 37.030066 | 4.455969 | 8.31 | < 2e-16 | *** |
| PDO | -20.662656 | 10.072626 | -2.051 | 0.040231 | * |
| PDO² | -24.104172 | 5.073734 | -4.751 | 0.000002 | *** |
| NPGO | 26.93484 | 11.121125 | 2.422 | 0.015437 | * |
| NPGO² | -37.855333 | 5.35912 | -7.064 | 0.000000 | *** |
| UI 1-mo lag | -23.52849 | 5.371519 | -4.38 | 0.000012 | *** |
| UI 1-mo lag² | 11.616668 | 3.947831 | 2.943 | 0.003255 | ** |
| year (2005):contour index | -0.002456 | 0.006158 | -0.399 | 0.689981 |  |
| year (2006):contour index | -0.014081 | 0.00758 | -1.858 | 0.063199 | . |
| year (2007):contour index | 0.020138 | 0.008216 | 2.451 | 0.014238 | * |
| year (2008):contour index | -0.028914 | 0.006515 | -4.438 | 0.000009 | *** |
| year (2009):contour index | -0.012649 | 0.007519 | -1.682 | 0.092525 | . |
| year (2010):contour index | -0.035252 | 0.006465 | -5.453 | 0.000000 | *** |
| year (2011):contour index | -0.032566 | 0.008179 | -3.982 | 0.000068 | *** |
| year (2012):contour index | -0.026658 | 0.008918 | -2.989 | 0.002797 | ** |
| year (2013):contour index | 0.00477 | 0.007963 | 0.599 | 0.549130 |  |
| year (2014):contour index | -0.029917 | 0.007531 | -3.973 | 0.000071 | *** |
| year (2015):contour index | -0.013494 | 0.01116 | -1.209 | 0.226602 |  |
| year (2016):contour index | -0.037838 | 0.009151 | -4.135 | 0.000036 | *** |
| year (2017):contour index | 0.001641 | 0.008002 | 0.205 | 0.837467 |  |
| Log(theta) | -1.168082 | 0.037784 | -30.915 | < 2e-16 | *** |
|  |  |  |  |  |  |
| Zero hurdle model coefficients (binomial with logit link): | | | |  |  |
|  | **Estimate** | **Std. Error** | **z value** | **Pr(>\|z\|)** |  |
| (Intercept) | 22.74339 | 12.18779 | 1.866 | 0.062030 | . |
| year (2005) | 3.63193 | 0.53429 | 6.798 | 0.000000 | *** |
| year (2006) | 0.90023 | 0.29543 | 3.047 | 0.002310 | ** |
| year (2007) | 0.09529 | 0.39093 | 0.244 | 0.807412 |  |
| year (2008) | 3.28542 | 0.85982 | 3.821 | 0.000133 | *** |
| year (2009) | 0.04671 | 0.3293 | 0.142 | 0.887190 |  |
| year (2010) | 1.94929 | 0.58746 | 3.318 | 0.000906 | *** |
| year (2011) | 0.9965 | 0.68521 | 1.454 | 0.145866 |  |
| year (2012) | 3.87067 | 0.98042 | 3.948 | 0.000079 | *** |
| year (2013) | -0.99096 | 0.37312 | -2.656 | 0.007910 | ** |
| year (2014) | 0.61935 | 0.33156 | 1.868 | 0.061764 | . |
| year (2015) | 1.1882 | 0.67881 | 1.75 | 0.080044 | . |
| year (2016) | 0.90338 | 0.4309 | 2.096 | 0.036040 | * |
| year (2017) | 3.04939 | 0.42593 | 7.159 | 0.000000 | *** |
| month | -0.61994 | 0.4276 | -1.45 | 0.147116 |  |
| month² | 0.05134 | 0.02955 | 1.737 | 0.082314 | . |
| Midwater Temperature | -17.06839 | 9.10103 | -1.875 | 0.060733 | . |
| Midwater Temperature² | 37.9703 | 5.51926 | 6.88 | 0.000000 | *** |
| Midwater Fluorescence | 0.12642 | 0.01764 | 7.168 | 0.000000 | *** |
| Midwater Salinity | -0.79564 | 0.36437 | -2.184 | 0.028991 | * |
| distance to 200 m | 10.32529 | 6.40289 | 1.613 | 0.106832 |  |
| distance to 200 m² | 9.42786 | 3.76827 | 2.502 | 0.012353 | * |
| distance to land | 20.11001 | 6.31886 | 3.183 | 0.001460 | ** |
| distance to land² | -13.37068 | 4.28481 | -3.12 | 0.001806 | ** |
| distance to island | -1.91968 | 3.58916 | -0.535 | 0.592751 |  |
| distance to island² | 7.22871 | 3.07539 | 2.351 | 0.018748 | * |
| contour index | 8.80382 | 4.36971 | 2.015 | 0.043932 | * |
| contour index² | 10.62592 | 3.60514 | 2.947 | 0.003204 | ** |
| SOI | -4.06537 | 10.07937 | -0.403 | 0.686701 |  |
| SOI² | 61.63123 | 10.83696 | 5.687 | 0.000000 | *** |
| PDO | 97.4962 | 15.39397 | 6.333 | 0.000000 | *** |
| PDO² | -33.57325 | 6.26452 | -5.359 | 0.000000 | *** |
| NPGO | 41.86046 | 17.05261 | 2.455 | 0.014097 | * |
| NPGO² | -39.02657 | 7.50628 | -5.199 | 0.000000 | *** |
| UI 3-mo lag | 19.78013 | 9.08013 | 2.178 | 0.029377 | * |
| UI 3-mo lag² | -33.65814 | 5.95769 | -5.65 | 0.000000 | *** |
