# Supplementary material for: Modeling predator and prey hotspots: Management implications of baleen whale co-occurrence with krill in Central California
Source: PLoS One. 2020 Jul 7;15(7):e0235603. doi: 10.1371/journal.pone.0235603 (PMC7340285; doi:10.1371/journal.pone.0235603)
Supplement: S4 Table — (DOCX) [file pone.0235603.s011.docx]

**Table 4.** Coefficients, standard errors, z values and p values for all quantitative variables (including years) for the negative binomial regression model for blue whales.

|  | **Estimate** | **Std. Error** | **z value** | **Pr(>\|z\|)** |  |
| --- | --- | --- | --- | --- | --- |
| Intercept | -41.197711 | 5.896002 | -6.9870 | 0.000000 | *** |
| year (2005) | 0.695067 | 0.923212 | 0.7530 | 0.451523 |  |
| year (2006) | -37.031168 | 4480490.7 | 0.0000 | 0.999993 |  |
| year (2007) | -4.96484 | 2.443384 | -2.0320 | 0.042158 | * |
| year (2008) | -34.958629 | 2332670.7 | 0.0000 | 0.999988 |  |
| year (2009) | -4.655214 | 1.153051 | -4.0370 | 0.000054 | *** |
| year (2010) | -1.748586 | 0.985798 | -1.7740 | 0.076100 | . |
| year (2011) | -5.581453 | 1.273001 | -4.3840 | 0.000012 | *** |
| year (2012) | -4.182248 | 1.094339 | -3.8220 | 0.000133 | *** |
| year (2013) | 0.325074 | 1.010156 | 0.3220 | 0.747600 |  |
| year (2014) | -0.606647 | 1.011863 | -0.6000 | 0.548816 |  |
| year (2015) | 3.146037 | 0.841678 | 3.7380 | 0.000186 | *** |
| year (2016) | 1.416984 | 0.797196 | 1.7770 | 0.075493 | . |
| year (2017) | 1.655783 | 0.808896 | 2.0470 | 0.040661 | * |
| month | 10.023023 | 1.572521 | 6.3740 | 0.000000 | *** |
| month² | -0.626313 | 0.09611 | -6.5170 | 0.000000 | *** |
| Surface Temperature | -36.822408 | 18.297258 | -2.0120 | 0.044172 | * |
| Surface Temperature² | -89.076674 | 22.958255 | -3.8800 | 0.000104 | *** |
| UI 3-mo lag | -0.011875 | 0.003208 | -3.7020 | 0.000214 | *** |
| SOI 3-mo lag | 0.331505 | 0.130156 | 2.5470 | 0.010866 | * |
| Surface Fluorescence | 20.284471 | 12.964693 | 1.5650 | 0.117678 |  |
| Surface Fluorescence² | 0.370508 | 9.946119 | 0.0370 | 0.970284 |  |
| Surface Fluorescence³ | -20.216341 | 10.66794 | -1.8950 | 0.058085 | . |
| PDO | -0.81877 | 0.360282 | -2.2730 | 0.023052 | * |
| average depth | -43.764854 | 16.760793 | -2.6110 | 0.009024 | ** |
| average depth² | -71.152554 | 22.95249 | -3.1000 | 0.001935 | ** |
| Midwater Salinity | 85.729071 | 28.404519 | 3.0180 | 0.002543 | ** |
| Midwater Salinity² | -32.292893 | 26.00969 | -1.2420 | 0.214395 |  |
| Midwater Salinity³ | 77.840288 | 23.283591 | 3.3430 | 0.000828 | *** |
| distance to 200 m | -0.040925 | 0.069465 | -0.5890 | 0.555760 |  |
| distance to 200 m² | -0.004639 | 0.001429 | -3.2470 | 0.001166 | ** |
| year (2005):distance to 200 m | -0.536205 | 0.285198 | -1.8800 | 0.060092 | . |
| year (2006):distance to 200 m | 0.093793 | 385981.47 | 0.0000 | 1.000000 |  |
| year (2007):distance to 200 m | 0.1907 | 0.146512 | 1.3020 | 0.193054 |  |
| year (2008):distance to 200 m | 0.055511 | 226379.7 | 0.0000 | 1.000000 |  |
| year (2009):distance to 200 m | 0.310499 | 0.088121 | 3.5240 | 0.000426 | *** |
| year (2010):distance to 200 m | 0.095926 | 0.076746 | 1.2500 | 0.211332 |  |
| year (2011):distance to 200 m | 0.139148 | 0.080083 | 1.7380 | 0.082289 | . |
| year (2012):distance to 200 m | 0.18487 | 0.07525 | 2.4570 | 0.014020 | * |
| year (2013):distance to 200 m | 0.032262 | 0.08814 | 0.3660 | 0.714341 |  |
| year (2014):distance to 200 m | 0.07495 | 0.086659 | 0.8650 | 0.387103 |  |
| year (2015):distance to 200 m | 0.004341 | 0.075266 | 0.0580 | 0.954005 |  |
| year (2016):distance to 200 m | 0.109236 | 0.073172 | 1.4930 | 0.135476 |  |
| year (2017):distance to 200 m | 0.092483 | 0.075413 | 1.2260 | 0.220066 |  |
