# Supplementary material for: Modeling predator and prey hotspots: Management implications of baleen whale co-occurrence with krill in Central California
Source: PLoS One. 2020 Jul 7;15(7):e0235603. doi: 10.1371/journal.pone.0235603 (PMC7340285; doi:10.1371/journal.pone.0235603)
Supplement: S5 Table — (DOCX) [file pone.0235603.s012.docx]

**Table 5.** Coefficients, standard errors, z values and p values for all quantitative variables (including years) for the negative binomial regression model for humpback whales.

|  | **Estimate** | **Std. Error** | **z value** | **Pr(>\|z\|)** |  |
| --- | --- | --- | --- | --- | --- |
| Intercept | -14.7532882 | 1.891959 | -7.79800 | 0.00000 | *** |
| year (2005) | -0.5676604 | 0.4506144 | -1.26000 | 0.20776 |  |
| year (2006) | -1.2749749 | 0.3878363 | -3.28700 | 0.00101 | ** |
| year (2007) | 0.8663867 | 0.3289617 | 2.63400 | 0.00845 | ** |
| year (2008) | 0.6789665 | 0.5027092 | 1.35100 | 0.17682 |  |
| year (2009) | -0.1425805 | 0.3622385 | -0.39400 | 0.69387 |  |
| year (2010) | 0.9611572 | 0.36673 | 2.62100 | 0.00877 | ** |
| year (2011) | -1.4783222 | 0.4720325 | -3.13200 | 0.00174 | ** |
| year (2012) | 0.9815432 | 0.4344239 | 2.25900 | 0.02386 | * |
| year (2013) | -0.1193904 | 0.4077015 | -0.29300 | 0.76965 |  |
| year (2014) | 1.2545352 | 0.436847 | 2.87200 | 0.00408 | ** |
| year (2015) | 1.699337 | 0.4805926 | 3.53600 | 0.00041 | *** |
| year (2016) | 2.2799577 | 0.4276117 | 5.33200 | 0.00000 | *** |
| year (2017) | 2.541497 | 0.3159445 | 8.04400 | 0.00000 | *** |
| month | 2.8868533 | 0.548152 | 5.26700 | 0.00000 | *** |
| month² | -0.1472531 | 0.0360234 | -4.08800 | 0.00004 | *** |
| Surface Fluorescence | -5.5614825 | 5.3839805 | -1.03300 | 0.30162 |  |
| Surface Fluorescence² | 4.1229762 | 3.9199798 | 1.05200 | 0.29290 |  |
| Surface Fluorescence³ | -10.100438 | 3.9186783 | -2.57800 | 0.00995 | ** |
| Midwater Fluorescence | 30.902582 | 5.5666226 | 5.55100 | 0.00000 | *** |
| Midwater Fluorescence² | -1.8137929 | 5.1578499 | -0.35200 | 0.72510 |  |
| Midwater Fluorescence³ | 1.6827993 | 3.7953359 | 0.44300 | 0.65749 |  |
| Midwater Fluorescence⁴ | -11.2777699 | 3.8562658 | -2.92500 | 0.00345 | ** |
| Surface Temperature | 15.6715472 | 8.1837692 | 1.91500 | 0.05550 | . |
| Surface Temperature² | -47.5237462 | 6.4630861 | -7.35300 | 0.00000 | *** |
| Midwater Temperature | -17.4258418 | 11.2438368 | -1.55000 | 0.12119 |  |
| Midwater Temperature² | 27.2134867 | 5.8123965 | 4.68200 | 0.00000 | *** |
| Surface Salinity | 15.5836487 | 6.2389763 | 2.49800 | 0.01250 | * |
| Surface Salinity² | 14.7184206 | 7.280738 | 2.02200 | 0.04322 | * |
| Surface Salinity³ | 24.0536168 | 7.4144621 | 3.24400 | 0.00118 | ** |
| Midwater Salinity | 3.2663408 | 9.5486557 | 0.34200 | 0.73230 |  |
| Midwater Salinity² | -22.1172923 | 6.2006442 | -3.56700 | 0.00036 | *** |
| average depth | -0.0007386 | 0.0002594 | -2.84800 | 0.00441 | ** |
| distance to 200 m | -0.060679 | 0.00646 | -9.39300 | > 2e -16 | *** |
| distance to island | -9.5537067 | 3.828356 | -2.49600 | 0.01258 | * |
| distance to island² | -13.7774931 | 3.7099388 | -3.71400 | 0.00020 | *** |
| UI 3-mo lag | -0.0078286 | 0.0013163 | -5.94800 | 0.00000 | *** |
| PDO 2-mo lag | -0.3718395 | 0.1502378 | -2.47500 | 0.01332 | * |
| NPGO 1-mo lag | -0.4892795 | 0.2142466 | -2.28400 | 0.02239 | * |
| SOI 3-mo lag | 0.1409161 | 0.0429472 | 3.28100 | 0.00103 | ** |
